# Cardiorenal outcomes with sodium/glucose cotransporter-2 inhibitors in patients with type 2 diabetes and low kidney risk: real world evidence

**DOI:** 10.1186/s12933-021-01362-y

**Published:** 2021-08-18

**Authors:** Meir Schechter, Cheli Melzer-Cohen, Aliza Rozenberg, Ilan Yanuv, Gabriel Chodick, Avraham Karasik, Mikhail Kosiborod, Ofri Mosenzon

**Affiliations:** 1 Faculty of Medicine, Hebrew University of Jerusalem, Jerusalem, Israel; 2 Diabetes Unit, Department of Endocrinology and Metabolism, Hadassah Medical Center, Jerusalem, Israel; 3 Maccabi Institute for Research and Innovation, Maccabi Healthcare Services, Tel-Aviv, Israel; 4 School of Public Health Sackler, Faculty of Medicine, Tel Aviv University, Tel Aviv, Israel; 5 Tel Aviv University, Tel Aviv, Israel; 6 Saint Luke’s Mid America Heart Institute, University of Missouri-Kansas City, Kansas, MO USA; 7 The George Institute for Global Health and University of New South Wales, Sydney, NSW Australia; 8 The Diabetes Unit, Department of Endocrinology and Metabolism, Hadassah Ein Kerem Medical Center, P.O.B 12000, 9112001 Jerusalem, Israel

**Keywords:** SGLT2i, Cardiorenal outcomes, Real world evidence, Type 2 diabetes

## Abstract

**Background:**

Randomized controlled trials showed that sodium/glucose cotransporter-2 inhibitors (SGLT2i) protect the heart and kidney in an array of populations with type 2 diabetes (T2D) and increased cardiorenal risk. However, the extent of these benefits also in lower kidney-risk T2D populations needs further investigation.

**Methods:**

Members of Maccabi Healthcare Systems listed in their T2D registry who initiated new glucose lowering agents (GLA), were divided into SGLT2i initiators and other GLAs (oGLAs). Groups were propensity score-matched by baseline demographic and medical characteristics. Two composite cardiovascular outcomes were defined: all-cause mortality (ACM) or hospitalization for heart failure (hHF); and ACM, myocardial infraction (MI) or stroke. The cardiorenal outcome was: ACM, new end-stage kidney disease (ESKD) or  ≥  40% reduction from baseline estimated glomerular filtration rate (eGFR). Renal-specific outcome was new ESKD or  ≥  40% eGFR reduction. Single components of cardiovascular and kidney outcomes were also assessed. Three subgroup definitions of low baseline kidney-risk were used: eGFR  >  90 ml/min/1.73 m^2^; urinary albumin below detectable levels; and low risk according to Kidney Disease: Improving Global Outcomes (KDIGO) classification. Analyses were performed utilizing an unadjusted model, and a model adjusted to baseline eGFR and urinary albumin-to-creatinine ratio.

**Results:**

Between April 1, 2015 and June 30, 2018; 68,187 patients initiated new GLAs — 11,321 SGLT2i initiators and 42,077 oGLAs initiators were eligible. Propensity score-matching yielded two comparable cohorts; each included 9219 participants. Median follow-up was 1.7 years. Compared to oGLAs, SGLT2i initiators had lower incidence of ACM or hHF [HR_95%CI_  =  0.62_(0.51–0.75)_]; ACM, MI or stroke [0.67_(0.57–0.80)_]; the cardiorenal outcome [0.65_(0.56–0.76)_]; and the renal-specific outcome [0.70_(0.57–0.85)_]. SGLT2i initiators also had lower risk for ACM, hHF and  ≥  30%,  ≥  40%,  ≥  50%,  ≥  57% eGFR reduction. No difference between groups was observed for MI or stroke. In the low baseline kidney-risk subgroups, SGLT2i initiation was generally associated with lower risk of the cardiovascular and cardiorenal outcomes, driven mainly by lower ACM incidence.

**Conclusions:**

Our findings in the general population of patients with T2D demonstrates lower risk of cardiorenal outcomes associated with initiation of SGLT2i compared with oGLAs, including specifically in patients with low baseline kidney-risk.

**Supplementary Information:**

The online version contains supplementary material available at 10.1186/s12933-021-01362-y.

## Introduction

Cardiovascular (CV) and kidney outcomes trials (CVOTs and KOTs) have shown that sodium/glucose cotransporter-2 inhibitors (SGLT2i) protect the heart and kidney in a variety of high-risk populations. In the context of type 2 diabetes (T2D) these benefits were shown in populations with established cardiovascular disease (CVD; EMPA-REG OUTCOME and to a lesser degree at VERTIS CV) [1–3] and/or with multiple CVD risk factors (CANVAS program, DECLARE-TIMI 58) [4–7]; chronic kidney disease (CKD; CREDENCE, SCORED, DAPA-CKD) [8–11], or with heart failure and reduced ejection fraction (HFrEF; DAPA-HF, EMPEROR-Reduced, SOLOIST-WHF) [12–15].

Based on these findings, recent position statements such as the 2020 Kidney Disease: Improving Global Outcomes (KDIGO) guidelines [16] and the 2021 American Diabetes Association (ADA) Standards of Care [17] recommended SGLT2i use in patients with T2D and increased risk for CKD, HFrEF and/or atherosclerotic CVD (AsCVD). An open question persists however, whether SGLT2i also exerts these protective effects in lower risk populations of patients with T2D, such as those with normal kidney markers, comprising most of the patients with T2D in the primary-care setting. This debate has practical clinical consequences—should SGLT2i be recommended to the general population of patients with T2D for the purpose of cardiorenal protection, independently of glycemic control?

In this observational study, we used the registry of Maccabi Healthcare Services (MHS), Israel’s second largest Health Maintenance Organization (HMO) insuring approximately 2.2 million subjects. Patients with T2D, who initiated a new glucose lowering agent (GLA) therapy between April 2015 and June 2018 were identified. SGLT2i initiators were propensity-scored matched with patients starting other GLAs (oGLAs), according to patients’ demographics, medical history, background medications and socioeconomic status. CV and kidney outcomes were analyzed in the entire population and in specific populations with low baseline kidney risk.

## Methods

### Study population

The study population was composed of patients registered in MHS Diabetes Registry [18] who initiated a new GLA treatment between April 1, 2015 and June 30, 2018. Index date was defined as the date of first filled prescription. Individuals with a previous prescription of that GLA class during the 365 days prior index date were not regarded as new users. Patients had to be  ≥  18-year-old with at least 1 year of data history in the MHS prior to index date. Only those with at least one eGFR measurement during the 180 days prior to the index date were included. Excluded were patients defined as type 1 diabetes in the MHS Diabetes Registry [18] or treated with insulin alone with no other GLA in the year prior to the index date. Also omitted were those with end-stage kidney disease (ESKD), on dialysis, or after kidney transplantation.

The sample population was also part of the main CVD-REAL 3 main report that compared kidney outcomes amongst SGLT2i initiators and oGLAs in five countries [19]. However, the protocol of this analysis has been adjusted for the data in the MHS database that has baseline urinary albumin-to-creatinine ratio (UACR) values for a remarkable portion of the participants, enabling specific focus on lower kidney-risk populations. Specifically, baseline UACR (and eGFR) values were used to develop the propensity-score model, and were adjusted to in the cox model. Specific subgroups of low-kidney risk patients were defined based on baseline UACR (and eGFR) values. To avoid immortal time bias [20, 21], the CVD-REAL 3 study included all episodes of GLA initiation during follow up. In the current analysis, to avoid this bias, each patient was included once, and only the first GLA initiation during the study period was defined as index date. The index medication was defined accordingly. In the main analysis patients who initiated a second GLA were kept in their original cohort. In a sensitivity analysis [‘strict on treatment’ (sOT) follow up definition], initiation of a second GLA resulted in follow-up termination (see below ‘follow up definition’).

The study received approval from MHS Institutional Review Board (IRB) committee at Bait Balev Hospital. Due to de-identified data extracting, informed consent was not requested by the IRB.

### Definitions of main variables

Laboratory measurements were performed in certified laboratories run by the MHS. eGFR was calculated using the Chronic Kidney Disease Epidemiology Collaboration (CKD-EPI) Equation [22], and the lower limit of detection of urinary albumin was 1.1 mg/dL. Baseline eGFR slope (per year) was calculated during the 4 years prior to the index date. Slope was calculated only for the participants with at-least 180 days interval between their first and their last (i.e., before index date) eGFR measurement during this period. All measurements were taken in a community setting, rather than during hospitalization, to reduce variations due to acute states, and defined as last evaluation within a year prior to the index date [except for baseline eGFR (180 days) and eGFR-slope calculation (4 years), as above]. Additional file 1: Table S1 presents the relevant ICD-9 (diagnosis) and ATC (medications) codes used in this study. History of established CVD, myocardial infarction (MI), heart failure (HF), stroke, transient ischemic attack (TIA), atrial fibrillation, hypertension and cancer were defined by inclusion in specific validated MHS registries [23–25] until the index date. Other co-morbidities were defined as having that diagnosis within a year prior to the index date. Baseline medications were defined as having at least one medication purchased during the year prior to index-date (not including index-date). Residential socioeconomic status (SES) was coded on a 1–10 scale developed by the Israeli Central Bureau of Statistics. This parameter was categorized into 4 groups (low [1–3], low-medium [4, 5], medium [6, 7] and high [8–10]).

### Follow up definitions

Patients were followed until migration, leaving the MHS, last date of data collection (set on June 30, 2018) or death date. The end of the intention to treat (ITT) period was defined by the last date of data collection or date of leaving the MHS (due to death or other reason), whichever came first. In a first sensitivity analysis, on treatment (OT) follow-up period was defined as the exposure time until last date of data collection, date of leaving the MHS or until treatment discontinuation—whichever came first. For this purpose, treatment discontinuation was defined as having a gap of more than 90 days, plus the treatment-period specified in the last prescription before treatment-cessation. In those that discontinued treatment, the follow-up ended once the number of days specified in the last prescription had passed, with the addition of 30-day grace period. In a second sensitivity analysis, a strict on treatment (sOT) follow-up period was defined. Criteria for sOT follow-up termination were those used for the OT definition, added by occasions of new GLA initiation. Analysis by the sOT definition was added to correct for possible biases associated with additional GLA(s) initiations (including SGLT2i’ in the oGLAs arm) during follow-up.

### Outcomes and subgroup definitions

Two composite CV outcomes were defined: (1) all-cause mortality (ACM) or hHF; and (2) ACM, stroke or MI. The cardiorenal outcome was defined as ACM, new ESKD or  ≥  40% reduction in eGFR from the last measurement prior the index date. The renal specific outcome included new ESKD or  ≥  40% reduction in eGFR. Single event outcomes were components of the composite outcome, as well as  ≥  30%,  ≥  50%, or  ≥  57% (i.e., doubling of serum creatinine) reduction in eGFR.

Incidence analyses of the study outcome in the matched cohort were conducted by treatment groups of the entire cohort and by baseline low kidney risk subgroups, defined in three different ways: (1) low KDIGO risk (UACR  <  30 mg/g and eGFR  >  60 ml/min/1.73 m^2^) [26]; (2) eGFR  >  90 ml/min/1.73 m^2^; (3) or urinary albumin below detectable levels (BDL). All patients had baseline eGFR values (i.e., 180 days prior the index date). Patients lacking baseline UACR values were included in the low KDIGO risk cohort, as long as they had eGFR  >  60 ml/min/1.73 m^2^.

### Statistical analysis

Propensity score was developed using a multivariate logistic regression model where the dependent binary variable indicated if the indexed medication was SGLT2i (= 1) or indexed medication was oGLA (= 0). Matching was generated on each eGFR layer separately (eGFR  >  90, 60–90 and  <  60 ml/min/1.73 m^2^). More than 40 demographics and medical covariates prior to treatment initiation were used for the propensity score. Continuous laboratory measurements were categorized, and a missing value was defined as “missing” category to allow all patients to be matched. All variable definitions are described in the supplementary methods. Briefly, amongst the included covariates were age, sex, index year, HbA1c, BMI, eGFR, UACR (below detectable levels (BDL),  <  30, 30–300 and  >  300 mg/g), diabetes and cardiovascular complications (based on diagnosis or MHS’s CVD registries [23–25]; Additional file 1: Table S1), glucose lowering agents, and cardiovascular or other medications.

Propensity score estimates the probability of initiating SGLT2i or other GLA given the covariates in the model. Patients were matched in 1:1 ratio by using greedy matching, which select SGLT2i treated patients and match the nearest oGLA-initiating subject. Caliper matching was defined as caliper  =  0.25 multiplied by the standard deviation of the propensity score distribution [27].

Patients’ demographic and medical characteristics were described at baseline period (prior to treatment initiation). Mean and standard deviation were used to describe continuous variables, while numbers of patients and percentages were used to describe categorical variables. Differences between patients who initiated SGLT2i or oGLA were assessed using standardized difference (STD). Significant difference in STD between groups was considered to be higher than 10%.

Only the first episode of the pre-defined cardiovascular or kidney event was included in the incidence analysis. Person-time at risk for each patient was the length of the index exposure episode, defined as the number of days from the day after the index prescription start date to the last day of follow-up. For each outcome of interest, the crude incidence rate in each index exposure group was the number of incident events divided by the total number of person-years at risk and was expressed per 100 person-years. The incidence rates for the SGLT2i group and the control group were then compared using a hazard ratio and corresponding 95% confidence interval. This analysis was performed using Cox proportional hazards regression and repeated in an unadjusted model and a model adjusted for baseline eGFR as a continuous variable and to baseline UACR as categorical variable (urine albumin BDL; UACR  >  0– <  30; 30– <  300;  ≥  300 mg/g).

In addition, these models were repeated within each of the low baseline kidney risk categories. Specifically for the analysis of the urinary albumin BDL category, the model was adjusted only by baseline eGFR.

### Role of the funding sources

No funding was received for this analysis.

## Results

### Study structure, baseline characteristics, and initiated medications

**Fig. 1:**
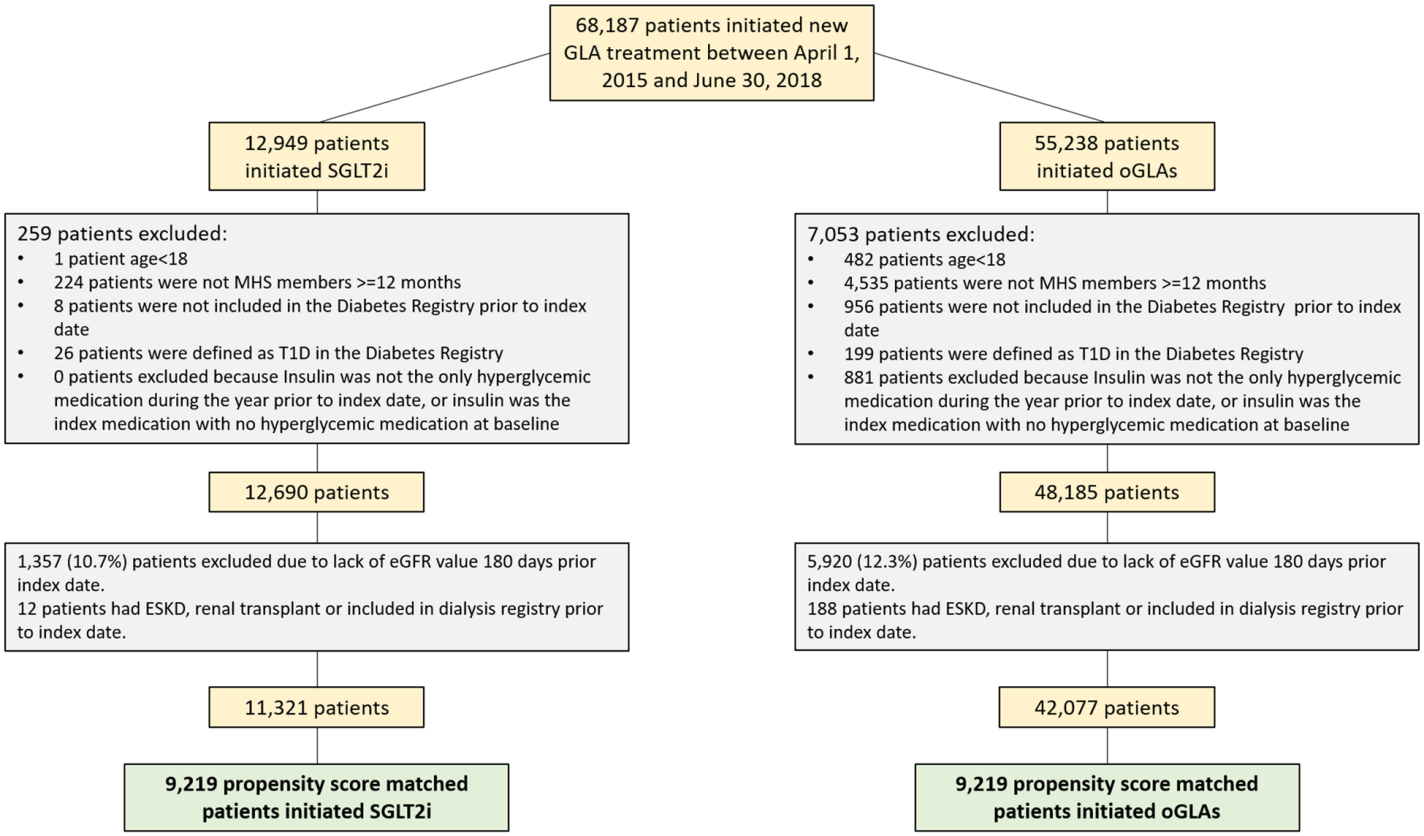
Participants flow chart. *GLA* glucose lowering agent; *SGLT2i* Sodium/glucose cotransporter-2 inhibitors; *ESKD* end stage kidney disease; *MHS* Maccabi Health Systems; *T1D* type 1 diabetes; *eGFR* estimated glomerular filtration rate

Between April 1st 2015 and June 30th 2018; 12,949 patients initiated treatment with SGLT2i and 55,238 patients initiated other GLAs (oGLAs). 11,321 SGLT2i initiators and 42,077 oGLAs initiators met the criteria to be included in this analysis (Fig. 1). Both groups were propensity score-matched according to their baseline demographic and clinical characteristics resulting in two comparable cohorts each included 9219 patients (Table 1, Additional file 1: Table S2). The population in this analysis included 39.7% women, mean (SD) age of 62.4 (10.3) years, most of them overweight (BMI  ≥  25 kg/m^2^) and 57.0% with diabetes duration longer than 10 years. Approximately 29.3% had established CVD history [24] and 19.5% had history of MI, coronary artery bypass grafting (CABG) or percutaneous coronary intervention (PCI) with stent. Mean (SD) baseline eGFR was 88.3 (18.5) ml/min/1.73 m^2^, mean annual eGFR slope was − 1.1 (2.7) mL/min/1.73 m^2^/year and median [IQR] baseline UACR was 13 (urine albumin BDL-49) mg/g. 11,768 (63.8%) had low KDIGO risk at baseline.

**Table 1:** Patients’ baseline characteristics post propensity-matching

Characteristic	Level	Study group		
		SGLT2-I (N = 9219)	oGLAs (N = 9219)	STD
Demographic characteristics				
Women	n (%)	3683 (40.0%)	3635 (39.4%)	0.01
Age (years)	Mean (SD)	62.3 (9.6)	62.5 (11.0)	− 0.01
Years in diabetes registry, n (%)	≤ 2	369 (4.0%)	362 (3.9%)	0.4
	2–5	1124 (12.2%)	1112 (12.1%)	
	5–10	2504 (27.2%)	2457 (26.7%)	
	> 10	5222 (56.6%)	5288 (57.4%)	
Socioeconomic status, n (%)	1–3 (low)	962 (10.4%)	881 (9.6%)	0.00
	4–5 (low-medium)	2825 (30.6%)	2930 (31.8%)	
	6–7 (medium)	3530 (38.3%)	3539 (38.4%)	
	8–10 (high)	1891 (20.5%)	1858 (20.2%)	
	Missing	11 (0.1%)	11 (0.1%)	
Baseline measures				
BMI	Mean (SD) in kg/m^2^	31.7 (5.4)	31.6 (5.4)	0.02
HbA1c (%)/mmol/mol	Mean (SD)	8.3 (1.5)/67.2 (2.3)	8.3 (1.6)/67.2(2.3)	− 0.04
eGFR (mL/min/1.73 m^2^), n (%)^a^	> 90 ml/min/1.73 m^2^	5026 (54.5%)	5026 (54.5%)	
	60–90 ml/min/1.73 m^2^	3407 (37.0%)	3407 (37.0%)	
	< 60 ml/min/1.73 m^2^	786 (8.5%)	786 (8.5%)	
UACR, n (%)	Urinary albumin BDL	3510 (38.1%)	3566 (38.7%)	0.06
	< 30 mg/g	2370 (25.7%)	2372 (25.7%)	
	30– < 300 mg/g	2312 (25.1%)	2295 (24.9%)	
	> 300 mg/g	664 (7.2%)	655 (7.1%)	
	Missing	363 (3.9%)	331 (3.6%)	
KDIGO risk [26], n (%)	Low-risk	5832 (63.3%)	5936 (64.4%)	0.05
	Moderate-risk	2439 (26.5%)	2250 (24.4%)	
	High and very high-risk	948 (10.3%)	1033 (11.2%)	
Change in eGFR, n (%)^b^	< 3 mL/min/1.73 m^2^/year	8757 (95.0%)	8645 (93.8%)	− 0.04
	≥ 3 mL/min/1.73 m^2^/year	358 (3.9%)	430 (4.7%)	
	≥ 5 mL/min/1.73 m^2^/year	110 (1.2%)	124 (1.3%)	− 0.01
	Missing	104 (1.1%)	144 (1.6%)	
Baseline medications				
Metformin	n (%)	8571 (93.0%)	8544 (92.7%)	0.01
Sulfonylureas	n (%)	2502 (27.1%)	2549 (27.6%)	− 0.01
DPP4i	n (%)	4402 (47.7%)	4373 (47.4%)	0.01
GLP-1 RA	n (%)	1806 (19.6%)	1880 (20.4%)	− 0.02
Metiglinides	n (%)	1093 (11.9%)	1074 (11.6%)	0.01
TZDs	n (%)	620 (6.7%)	667 (7.2%)	− 0.02
Acarbose	n (%)	210 (2.3%)	214 (2.3%)	− 0.00
Insulin	n (%)	2474 (26.8%)	2295 (24.9%)	0.04
Short-acting	n (%)	582 (6.3%)	557 (6.0%)	0.01
Long-acting	n (%)	2171 (23.5%)	2105 (22.8%)	0.02
ACEi/ARBs	n (%)	6595 (71.5%)	6436 (69.8%)	0.04
Beta blockers	n (%)	3664 (39.7%)	3634 (39.4%)	0.01
Aldosterone antagonists	n (%)	413 (4.5%)	415 (4.5%)	− 0.00
Medical history				
Established CVD history [24]	n (%)	2705 (29.3%)	2697 (29.3%)	0.00
Myocardial infarction/CABG/PCI with stent	n (%)	1795 (19.5%)	1796 (19.5%)	− 0.00
Microvascular complications^c^	n (%)	5624 (61.0%)	5495 (59.6%)	0.03
Heart failure (24)	n (%)	344 (3.7%)	307 (3.3%)	0.02

*ACEi* angiotensin-converting enzyme inhibitors; *ARBs* Angiotensin II receptor blocker; *BDL* below detectable levels; *CABG* coronary artery bypass grafting; *DPP4i* Dipeptidyl peptidase-4 inhibitor; *eGFR* estimated glomerular filtration rate; *GLP-1 RAs* glucagon-like peptide-1 receptor agonists; *KDIGO* Kidney Disease: Improving Global Outcome; *oGLA* other glucose lowering agent; *PCI* percutaneous coronary intervention; *STD* standardized difference; *TZD* Thiazolidinediones; *UACR* urinary albumin to creatinine ratio

^a^The propensity score matching was generated on each eGFR layer separately, therefore the number of participants in each arm's eGFR layer is equal by definition

^b^Baseline eGFR slope (per year) was calculated during the 4 years prior to the index date. Slope was calculated only for the participants with at-least 180 days interval between their first and their last (i.e., before index date) eGFR measurement during this period

^c^Microvascular complications was defined as: diabetic eye complication, neuropathy, diabetic foot/ peripheral angiopathy or diabetic kidney disease (i.e., nephropathy, eGFR  <  60 or UACR  >  100)

In the SGLT2i group, 34.0% of treatment initiations were with dapagliflozin and 66.0% were with empagliflozin. In the oGLAs group most patients initiated DPP4i (31.1%), metformin (18.2%), glucagon-like peptide-1 receptor agonists (GLP-1 RAs; 16.8%), insulin (11.1%), sulfonylurea (9.7%) or meglitinides (6.6%). Others initiated a regimen which included thiazolidinediones or acarbose. For the ITT follow up definition the mean exposure time was 1.7 (0.9) years. The distribution of each GLA within the cohorts, including the duration of follow up per each definition is presented in Additional file 1: Table S3.

### CV and kidney outcomes in the total cohort

**Fig. 2:**
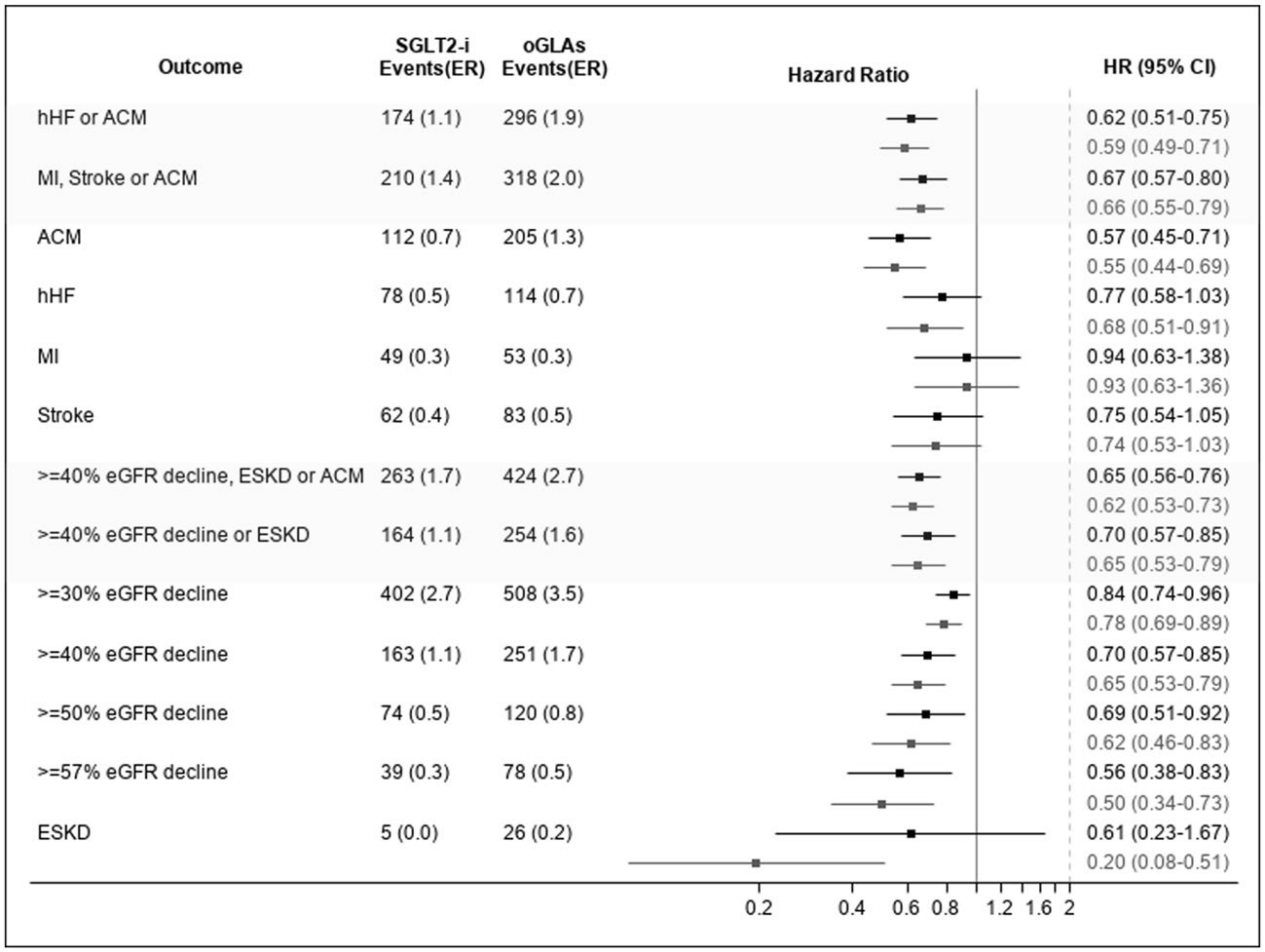
Risk for cardiovascular and kidney outcome in SGLT2i initiators compared to oGLAs in the entire cohort, during the ITT follow up definition. Event rates are presented as number of events per 100 person years of follow up. In black—the unadjusted model; and in grey—the model adjusted to baseline eGFR (as continuous variable) and UACR (as categorical variable). *SGLT2i* sodium/glucose cotransporter-2 inhibitors; *oGLAs* other glucose lowering agents; *ITT* intention to treat; *hHF* hospitalization for heart failure; *ACM* all-cause mortality; *MI* myocardial infract; *eGFR* estimated glomerular filtration rate; *ESKD* end stage kidney disease; *ER* event rate

Figure 2 presents the hazard ratios for the different CV and kidney outcomes in those initiating SGLT2i compared with oGLAs in the ITT follow-up definition. Generally consistent results were obtained in an unadjusted model and in a model adjusted to baseline UACR and eGFR. Those that initiated SGLT2i experienced lower event-rates of both CV composite outcomes: hHF or ACM [HR (95% CI)  =  0.62 (0.51–0.75)_adjusted_] as well as ACM, MI or stroke [HR (95% CI)  =  0.67 (0.57–0.80)_adjusted_]. These differences were mostly driven from lower risk of ACM in the SGLT2i cohort [HR (95% CI)  =  0.57 (0.45–0.71)_adjusted_], and for hHF [HR (95% CI) 0.77 (0.58–1.03)_adjusted_]. The event rate for stroke and MI were not significantly different between initiators of SGLT2i and oGLAs.

SGLT2i initiators had lower risk for adverse kidney events, including the composite cardiorenal [ACM, ESKD or  ≥  40% reduction in eGFR; HR (95% CI)  =  0.65 (0.56–0.76)_adjusted_] and renal specific outcomes [ESKD or  ≥  40% reduction in eGFR; HR (95% CI)  =  and 0.70 (0.57–0.85) _adjusted_]. The SGLT2i cohort had lower risk for most of the tested single kidney outcomes, except for the risk of new ESKD outcome, which was limited by small number of events, and in which adjustment to baseline kidney markers attenuated the between group differences [n_(SGLT2i)_  =  5 and n_(oGLAs)_  =  26; HR (95% CI)  =  0.20 (0.08–0.51)_unadjusted_ and 0.61 (0.23–1.67)_adjusted_].

### CV and kidney outcomes in populations of low kidney risk at baseline

The risk for adverse CV and kidney outcomes was also tested in the three different definitions of low baseline kidney risk subgroups: low KDIGO risk [UACR  <  30 mg/g and eGFR  >  60 ml/min/1.73 m^2^; 11,768 (63.8%) patients]; baseline eGFR  >  90 ml/min/1.73 m^2^ [10,052 (54.5%) patients]; urinary albumin BDL [7076 (38.4%) patients]. Due to lack of ESKD events in these low-risk subgroups, kidney outcomes were analyzed without new ESKD events. The presented results were obtained with the model adjusted to baseline kidney function; the unadjusted model yielded highly similar results.

**Fig. 3:**
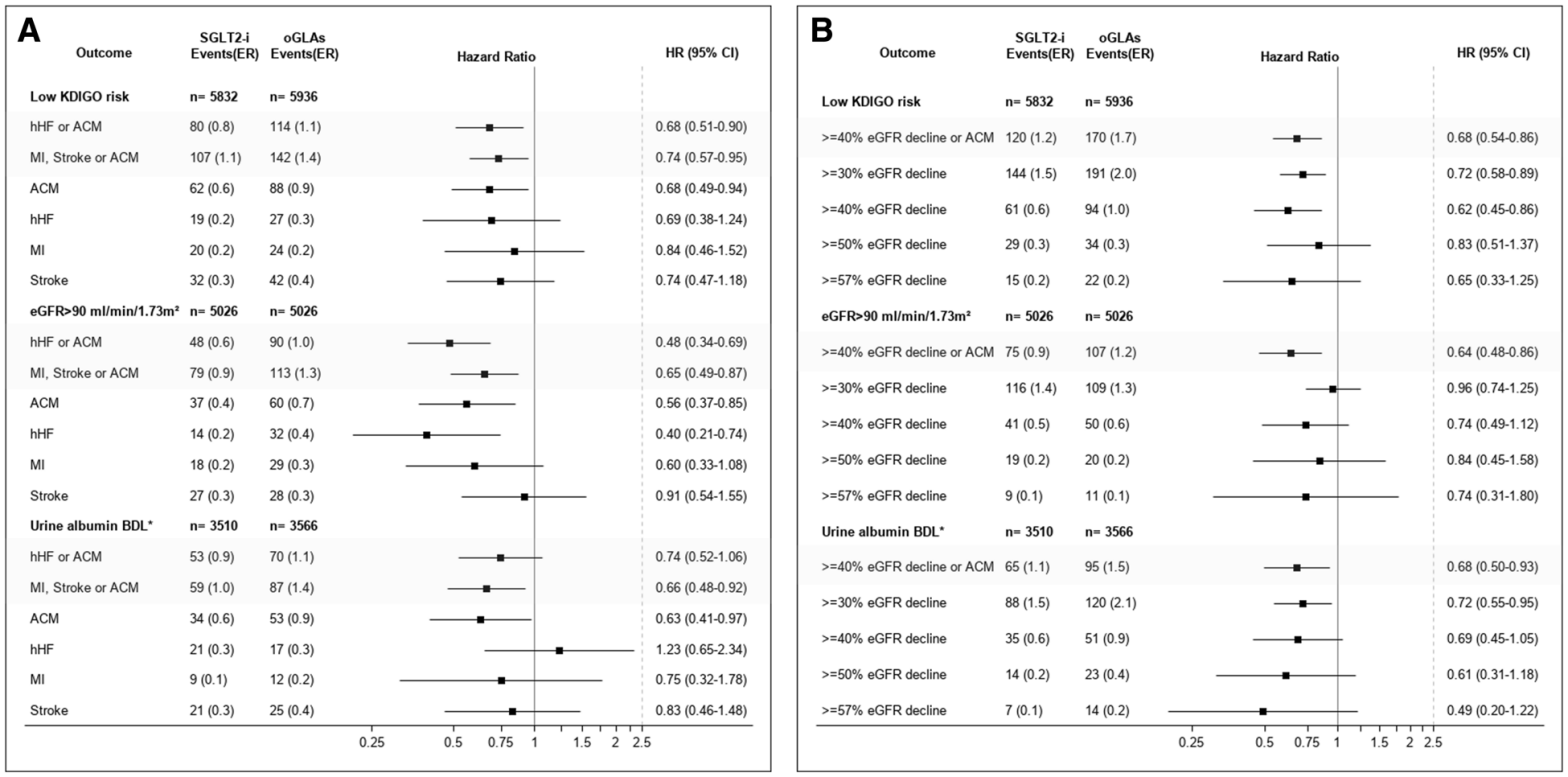
Risk for cardiovascular and kidney outcomes in SGLT2i initiators compared to oGLAs in low kidney risk populations, during the ITT follow up definition. **A** Cardiovascular outcomes. **B** Kidney outcomes. Event rates are presented as number of events per 100 person-years of follow up. Low KDIGO risk is defined as eGFR  >  60 ml/min/1.73 m^2^ and UACR  <  30 mg/g. For the low KDIGO risk and eGFR  >  90 ml/min/1.73 m^2^, the model was adjusted to baseline eGFR (as continuous variable) and UACR (as categorical variable). Outcome analysis of the urine albumin BDL category was only adjusted to baseline eGFR as continuous variable. *BDL  =  Below detectable levels. *SGLT2i* sodium/glucose cotransporter-2 inhibitors; *oGLAs* other glucose lowering agents; *ITT* intention to treat; *hHF* hospitalization for heart failure; *ACM* all-cause mortality; *MI* myocardial infract; *eGFR* estimated glomerular filtration rate; *UACR* urinary albumin to creatinine ratio; *KDIGO* kidney disease: improving global outcomes; *ER* event rate

Compared with oGLAs, initiation of SGLT2i was generally associated with lower event rates of the two composite cardiovascular outcomes as well as the cardiorenal outcome, across the different low kidney risk subgroups (Fig. 3). Specifically, the hazard ratio of the risk for the composite CV outcome of ACM, stroke or MI in SGLT2i initiators ranged between 0.67 and 0.76 in the low kidney risk subgroups. For the hHF or ACM composite outcome, SGLT2i initiation was associated with lower event rates in those with baseline low KDIGO risk and those with eGFR  >  90 ml/min/1.73 m^2^, but no statistical significance was observed in the group of baseline urinary albumin below detectable levels [HR (95% CI)  =  0.74 (0.52–1.06)] (Fig. 3A). The risk of composite cardiorenal outcome was consistently lower in SGLT2i initiators in all the tested low baseline kidney risk subgroups, with HR ranging between 0.69 and 0.72 (Fig. 3B).

Single component CV and kidney outcomes were also analyzed. While the risk for MI or stroke was not significantly different between groups, the incidence of ACM was lower in SGLT2i initiators across all low kidney risk subgroups. The risk of hHF was lower in SGLT2i initiators within the eGFR  >  90 ml/min/1.73 m^2^ subgroup, while no significant changes were observed in the other tested baseline kidney functions subgroups (Fig. 3A). The single kidney outcomes are presented in Fig. 3B.

Analysis of the CV and kidney outcomes by OT and sOT follow up definitions showed generally consistent results. The outcomes for the entire cohort are presented in Additional file 2: Figure S1, Additional file 5: FigureS3 and for the low baseline kidney risk subgroups in Additional files 3, 4: Figure S2, Additional files 6, 7: Figure S4, for the OT and sOT definitions, respectively.

## Discussion

In this observational study, patients with T2D who initiated SGLT2i were propensity-score matched with those who initiated oGLAs. SGLT2i initiation was associated with improvement in two tested composite CV outcomes; [1] ACM, MI or stroke; [2] ACM or hHF, as well as in the cardiorenal and the renal specific composite outcomes. SGLT2i initiators also experienced lower event rates of most of the single outcomes: ACM, hHF, eGFR reduction from baseline of  ≥  30%,  ≥  40%,  ≥  50% or  ≥  57%. The incidence of stroke or MI was not significantly different between the groups across all follow-up definitions. Importantly, similar trends were observed in subgroups defined by their low kidney risk at baseline: low KDIGO risk; eGFR  >  90 ml/min/1.73 m^2^; and urinary albumin BDL. To the best of our knowledge, this is the first time that SGLT2i associated reduction in cardiorenal risk is shown in populations with urinary albumin BDL. Together these findings consolidate the CV and kidney protective role of SGLT2i in a real-world setting in comparison to other GLAs, and in populations with healthy kidney status.

Robust observational studies from well-phenotyped patient populations are a good data source for evaluations of low-risk patients, that are often excluded from RCTs. Several CVOTs (EMPA-REG OUTCOME [1, 2], CANVAS program [4], DECLARE-TIMI 58 [6] and VERTIS CV [3]) tested the CV safety and efficacy of SGLT2i in patients with T2D and increased CV risk. Other studies specifically focused on populations with T2D and CKD or HFrEF [8, 9, 14]. They generally found that compared with placebo SGLT2i protect the kidney and reduce the incidence of hHF [2–4, 6, 8–10, 12–14, 28, 29]. However, due to the relatively high baseline risk of the participants in these trials, external validity to the general T2D population has been questioned [30]. For example, the prevalence of established CVD in these CVOTs varied between 40.6% in the DECLARE-TIMI 58 trial [31], 65.6% in the CANVAS program [4], and the entire sample populations of EMPA-REG OUTCOME and VERTIS CV [1, 3]. In comparison, our propensity-matched cohort had only 29.3% baseline prevalence of established CVD (Table 1), similar to other cross-sectional reports of the general T2D population in different countries [30, 32, 33]. Kidney-wise, baseline mean eGFR of the participants in our analysis was 88.3 ml/min/1.73 m^2^ compared with 74.2–77.2 ml/min/1.73 m^2^ in the EMPA-REG OUTCOME, CANVAS program and VERTIS CV [2–4], and 85.3 ml/min/1.73 m^2^ in the DECLARE-TIMI 58 [6]. Baseline eGFR slope of this trial's subjects was − 1.1 ml/min/1.73 m^2^/year; only 4.3% had a baseline annual eGFR slope of  ≥  3 ml/min/1.73 m^2^/year (fast-decliners) and 1.2% of  ≥  5 ml/min/1.73 m^2^/year (severe-decliners). All in all, our sample population seems to have lower baseline kidney and CV risk than the participants in these CVOTs. Thus, the observed reduction in cardiorenal risk associated with SGLT2i initiation suggests that these benefits may apply to broader populations with T2D.

While associated risks for ACM or hHF were generally lower following treatment with SGLT2i, we did not find a significant reduction in the risk for MI and stroke (especially in the OT and sOT follow-up definitions). Such trends were observed in other CVOTs and other real-world evidence (RWE) [2–4, 6, 34]. Of note, in these reports SGLT2i reduce hHF episodes more overtly relative to ACM [6, 28]. Here, however, in the low baseline kidney risk categories ACM incidence was consistently lower in SGLT2i initiators, while the risk for hHF was not stable, i.e., significant between group differences were observed only in the eGFR  >  90 ml/min/1.73 m^2^ category. Unlike ACM, hHF is a clinical diagnosis that relies on physicians’ reporting, introducing a limitation to the interpretation of this outcome in RWE settings. Importantly, hHF event, as well as ESKD, rarely occur in patients with healthy kidney markers, precluding the capture of outcome differences in low kidney risk populations [35].

The cardiorenal improvement seen here with SGLT2i treatment has been similarly documented in other RWE studies [19, 34, 36–40]. The sample used for this study has been part of the CVD-REAL 3 study [19] that found better kidney outcomes in SGLT2i initiators compared to oGLAs across five countries. Here we used slightly different follow up definitions, further consolidating the results of the CVD-REAL 3, using a complementary approach (see “Methods” Section). While CVD-REAL 3 mainly focused on kidney outcomes, here we also tested CV outcomes. Importantly, the cardiorenal risk associated with SGLT2i initiation compared to oGLAs was specifically tested in different populations defined by their low baseline kidney risk. The current analysis was designed to emphasize the strengths of the MHS database. Additional variables were introduced into the propensity score matching, such as patients’ SES information and baseline UACR. Presence of UACR values for most of the participants (96.5%), provided a unique opportunity to analyze populations defined as low-kidney risk by both baseline eGFR and UACR. Specifically, we found SGLT2i-associated improvement in the cardiorenal outcome even in those with urinary albumin BDL, a relatively novel kidney definition based on cumulative results that any urinary albumin excretion, even within the normoalbuminuric range, is associated with worse outcomes [41–45]. Relevantly, SGLT2i were reported to improve albuminuria status—serving as a surrogate for kidney decline and a possible mediator– even in patients with T2D and normal kidney markers [5, 45, 46]. These results suggest a beneficial role for SGLT2i early in the disease process.

According to the 2021 ADA Standards of Care, SGLT2i are indicated for cardiorenal protection purposes in patients with T2D and CKD, HF or atherosclerotic cardiovascular disease (AsCVD)—independent of glycemic control or previous metformin use [17]. Similarly, the KDIGO guidelines recommend to treat most patients with T2D and CKD with SGLT2i [16]. A controversy persists whether SGLT2i have a cardiorenal preventive role in patients with T2D and normal kidney markers. In this analysis, the lower incidence of the composite CV and cardiorenal outcomes was generally conserved in the different low baseline kidney risk categories, with some variations. Importantly, lower risk for ACM was observed in the defined low baseline kidney risk categories. However, variation of the HR values for the other single component outcomes in this population precludes a specific conclusion. Thus, although our findings support a cardiorenal protective role of SGLT2i in patients with T2D and low baseline kidney risk, longer follow up on more participants may be required for more definitive answers.

Recent years have brought a renaissance in the treatment of diabetes kidney disease (DKD). The gold standard of risk factor modifications and angiotensin-converting enzyme inhibitors/angiotensin II receptor blockers (ACEi/ARBs) [47–49] is now joined by SGLT2i therapy. Finerenone, a non-steroidal mineralocorticoid receptor antagonist (MRA), improved kidney outcomes in patients with DKD (the FIDELIO-DKD trial [50], and an ongoing study tests CV outcome (The FIGARO-DKD trial, NCT02545049). Cumulative evidence indicate that GLP-1 RAs may also have a kidney protective role [38, 51, 52], and the FLOW KOT [NCT03819153 (53)] is expected to provide a more definite answer. The SONAR trial indicated that specific patients with DKD could benefit from endothelin receptor antagonists (ERA) [54]. However, to date no medications have been approved for DKD prevention purposes in patients with T2D and healthy kidney markers. Our findings of lower cardiorenal risk following SGLT2i initiation, as well as recent post-hoc analyses from RCTs and other RWE, suggest that SGLT2i may exert such a role [7, 40, 52].

This study enjoys several strengths. In the final matched-cohort 3.5% lacked UACR measurement and 1.1% did not have a calculated eGFR slope at baseline. The completeness of the data, including medical history, background medication, and socioeconomic status, has allowed the formation of comparable cohorts. Baseline eGFR slope was not included in the propensity-score and yet was comparable in both arms, testifying for the relatively balanced nature of the cohorts. The relatively large proportion of patients with low baseline kidney risk enabled meaningful results in these specific subgroups of interest.

The study also has several limitations. First and foremost, treatment allocation was not assigned in a randomized controlled manner. It is possible that unknown confounders, that were not part of the propensity-matching, influenced the study outcome. Initiation of other GLAs or other cardiovascular or kidney medications (e.g., GLP-1 RAs or ACEi/ARBs amongst others) following the index date was also not accounted for. Baseline allocation of UACR and eGFR values were based on a single measurement, although these markers have some day-to-day variability. Another limitation is the lack of data regarding treatment adherence and specific changes in GLAs regimen during follow up. The risk for each outcome was therefore calculated for ITT, OT and sOT follow-up definitions; each has its own advantages and limitations, with generally similar findings. Lack of prospective data collection also affected the outcome definitions. Our registry does not specify the cause of death, precluding us from testing the association between SGLT2i initiations and CV death, as a component of the classical major adverse cardiovascular event (MACE) composite outcome, amongst other possible causes of death. Relevantly, several SGLT2i randomized controlled trials (RCTs) and RWEs have found lower incidence of ACM, suggesting an effect that may extend beyond MACE (EMPA-REG OUTCOME, DAPA-CKD) [1, 10, 28, 34]. Other outcomes (e.g., hHF) may be affected by variations between physicians’ reporting. Finally, we focused on populations with low baseline kidney risk, however we did not test stricter low baseline risk definitions e.g., those lacking CVD—this question should be analyzed in larger cohorts with longer follow up.

## Conclusion

In conclusion, this observational, propensity-score matched analysis demonstrates that initiation of SGLT2i treatment  in patients with T2D is associated with lower risk for adverse cardiorenal outcomes. These findings were generally consistent in populations of patients defined by their lower baseline kidney risk.

## Supplementary Information

**Additional file 1: Table S1.** Variable’s definitions, including medications and diagnosis. *ACE* – Angiotensin-converting enzyme; *ARB* – Angiotensin II receptor blocker; *CABG* – coronary artery bypass grafting; *CVD* – cardiovascular disease; *DPP4* – Dipeptidyl-peptidase 4; *eGFR* – estimated glomerular filtration rate; *GLP-1 RA* – glucagon-like peptide-1 receptor agonist; *ICD-9* – International Classification of Diseases 9; *MHS* – Maccabi Healthcare Services; *PCI* – percutaneous coronary intervention. **Table S2.** Additional patients’ baseline characteristics post propensity-matching. **Table S3.** Distribution of index medications post-match and by follow up definitions. *PP4i* – Dipeptidyl peptidase-4 inhibitor; *GLP1-RA* – Glucagon-like peptide-1 receptor agonists; *ITT* – intention to treat; *oGLAs* – other glucose lowering agents; *OT* – on treatment; *SGLT2i* – Sodium-glucose cotransporter 2 inhibitors; *sOT* – strict on treatment; *TZDs* – Thiazolidinediones.

**Additional file 2:Figure S1.** Risk for cardiovascular and kidney outcome in SGLT2i initiators compared to oGLAs in the entire cohort, during the OT follow up definition. Event rates are presented as number of events per 100 person years of follow up. In black—the unadjusted model; and in grey—the model adjusted to baseline eGFR (as continuous variable) and UACR (as categorical variable). SGLT2i = sodium/glucose cotransporter-2 inhibitors; oGLAs = other glucose lowering agents; OT = on treatment; hHF = hospitalization for heart failure; ACM =all-cause mortality; MI = myocardial infract; eGFR = estimated glomerular filtration rate; ESKD = end stage kidney disease; ER = event rate.

**Additional file 3: Figure S2.** Risk for cardiovascular and kidney outcomes in SGLT2i initiators compared to oGLAs in low kidney risk populations, during the OT follow up definition. A Cardiovascular outcomes. B Kidney outcomes. Event rates are presented as number of events per 100 person-years of follow up. Low KDIGO risk is defined as eGFR>60 ml/min/1.73 m^2^ and UACR<30 mg/g. For the low KDIGO risk and eGFR>90 ml/min/1.73 m^2^, the model was adjusted to baseline eGFR (as continuous variable) and UACR (as categorical variable). Outcome analysis of the urine albumin BDL category was only adjusted to baseline eGFR as continuous variable. * BDL= Below detectable levels. SGLT2i = sodium/glucose cotransporter-2 inhibitors; oGLAs = other glucose lowering agents; OT = on treatment; hHF = hospitalization for heart failure; ACM =all-cause mortality; MI = myocardial infract; eGFR = estimated glomerular filtration rate; UACR = urinary albumin to creatinine ratio; KDIGO = kidney disease: improving global outcomes; ER =event rate.

**Additional file 4: Figure S2.** Risk for cardiovascular and kidney outcomes in SGLT2i initiators compared to oGLAs in low kidney risk populations, during the OT follow up definition. A Cardiovascular outcomes. B Kidney outcomes. Event rates are presented as number of events per 100 person-years of follow up. Low KDIGO risk is defined as eGFR>60 ml/min/1.73 m^2^ and UACR<30 mg/g. For the low KDIGO risk and eGFR>90 ml/min/1.73 m^2^, the model was adjusted to baseline eGFR (as continuous variable) and UACR (as categorical variable). Outcome analysis of the urine albumin BDL category was only adjusted to baseline eGFR as continuous variable. * BDL= Below detectable levels. SGLT2i = sodium/glucose cotransporter-2 inhibitors; oGLAs = other glucose lowering agents; OT = on treatment; hHF = hospitalization for heart failure; ACM =all-cause mortality; MI = myocardial infract; eGFR = estimated glomerular filtration rate; UACR = urinary albumin to creatinine ratio; KDIGO = kidney disease: improving global outcomes; ER =event rate.

**Additional file 5: Figure S3.** Risk for cardiovascular and kidney outcome in SGLT2i initiators compared to oGLAs in the entire cohort, during the sOT follow up definition. Event rates are presented as number of events per 100 person years of follow up. In black—the unadjusted model; and in grey—the model adjusted to baseline eGFR (as continuous variable) and UACR (as categorical variable). SGLT2i = sodium/glucose cotransporter-2 inhibitors; oGLAs = other glucose lowering agents; sOT = strict on treatment; hHF = hospitalization for heart failure; ACM =all-cause mortality; MI = myocardial infract; eGFR = estimated glomerular filtration rate; ESKD = end stage kidney disease; ER = event rate.

**Additional file 6: Figure S4.** Risk for cardiovascular and kidney outcomes in SGLT2i initiators compared to oGLAs in low kidney risk populations, during the sOT follow up definition. A Cardiovascular outcomes. B Kidney outcomes. Event rates are presented as number of events per 100 person-years of follow up. Low KDIGO risk is defined as eGFR>60 ml/min/1.73 m^2^ and UACR<30 mg/g. For the low KDIGO risk and eGFR>90 ml/min/1.73 m^2^, the model was adjusted to baseline eGFR (as continuous variable) and UACR (as categorical variable). Outcome analysis of the urine albumin BDL category was only adjusted to baseline eGFR as continuous variable. *BDL= Below detectable levels. SGLT2i = sodium/glucose cotransporter-2 inhibitors; oGLAs = other glucose lowering agents; sOT = strict on treatment; hHF = hospitalization for heart failure; ACM =all-cause mortality; MI = myocardial infract; eGFR = estimated glomerular filtration rate; UACR = urinary albumin to creatinine ratio; KDIGO = kidney disease: improving global outcomes; ER =event rate.

**Additional file 7: Figure S4.** Risk for cardiovascular and kidney outcomes in SGLT2i initiators compared to oGLAs in low kidney risk populations, during the sOT follow up definition. A Cardiovascular outcomes. B Kidney outcomes. Event rates are presented as number of events per 100 person-years of follow up. Low KDIGO risk is defined as eGFR>60 ml/min/1.73 m^2^ and UACR<30 mg/g. For the low KDIGO risk and eGFR>90 ml/min/1.73 m^2^, the model was adjusted to baseline eGFR (as continuous variable) and UACR (as categorical variable). Outcome analysis of the urine albumin BDL category was only adjusted to baseline eGFR as continuous variable. *BDL= Below detectable levels. SGLT2i = sodium/glucose cotransporter-2 inhibitors; oGLAs = other glucose lowering agents; sOT = strict on treatment; hHF = hospitalization for heart failure; ACM =all-cause mortality; MI = myocardial infract; eGFR = estimated glomerular filtration rate; UACR = urinary albumin to creatinine ratio; KDIGO = kidney disease: improving global outcomes; ER =event rate.

## Data Availability

The datasets used and/or analysed during the current study are available from the corresponding author on reasonable request.

## Abbreviations

ACEi: Angiotensin-converting enzyme inhibitors
ACM: All-cause mortality
ARBs: Angiotensin II receptor blocker
AsCVD: Atherosclerotic cardiovascular disease
BDL: Below detectable levels
CABG: Coronary artery bypass grafting
CKD: Chronic kidney disease
CKD-EPI: Chronic Kidney Disease Epidemiology Collaboration
CV: Cardiovascular
CVD: Cardiovascular disease
CVOT: Cardiovascular outcomes trial
DKD: Diabetes kidney disease
eGFR: Estimated glomerular filtration rate
ERA: Endothelin receptor antagonist
ESKD: End-stage kidney disease
GLA: Glucose lowering agent
GLP-1 RAs: Glucagon-like peptide-1 receptor agonists
HF: Heart failure
hHF: Hospitalization for heart failure
HMO: Health Maintenance Organization
IRB: Institutional Review Board
ITT: Intention to treat
KDIGO: Kidney Disease Improving Global Outcome
KOT: Kidney outcomes trial
MACE: Major adverse cardiovascular event
MHS: Maccabi Healthcare Services
MI: Myocardial infraction
MRA: Mineralocorticoid receptor antagonist
oGLA: Other glucose lowering agent
PCI: Percutaneous coronary intervention
OT: On treatment
RCT: Randomized controlled trial
RWE: Real world evidence
SES: Socioeconomic status
SGLT2i: Sodium/glucose cotransporter-2 inhibitors
sOT: Strict on treatment
STD: Standardized difference
T2D: Type 2 diabetes
UACR: Urinary albumin to creatinine ratio
