# Supplementary material for: Cardiorenal outcomes with sodium/glucose cotransporter-2 inhibitors in patients with type 2 diabetes and low kidney risk: real world evidence
Source: Cardiovasc Diabetol. 2021 Aug 18;20:169. doi: 10.1186/s12933-021-01362-y (PMC8375057; doi:10.1186/s12933-021-01362-y)
Supplement: Supplementary file 1 — Additional file 1: Table S1. Variable’s definitions, including medications and diagnosis. ACE – Angiotensin-converting enzyme; ARB – Angiotensin II receptor blocker; CABG – coronary artery bypass grafting; CVD – cardiovascular disease; DPP4 – Dipeptidyl-peptidase 4; eGFR – estimated glomerular filtration rate; GLP-1 RA – glucagon-like peptide-1 receptor agonist; ICD-9 – International Classification of Diseases 9; MHS – Maccabi Healthcare Services; PCI – percutaneous coronary intervention. Table S2. Additional patients’ baseline characteristics post propensity-matching. Table S3. Distribution of index medications post-match and by follow up definitions. PP4i – Dipeptidyl peptidase-4 inhibitor; GLP1-RA – Glucagon-like peptide-1 receptor agonists; ITT – intention to treat; oGLAs – other glucose lowering agents; OT – on treatment; SGLT2i – Sodium-glucose cotransporter 2 inhibitors; sOT – strict on treatment; TZDs – Thiazolidinediones. [file 12933_2021_1362_MOESM1_ESM.docx]

**Cardiorenal outcomes with sodium/glucose cotransporter-2 inhibitors in patients with type 2 diabetes and low kidney risk: Real world evidence**

*Meir Schechter^1,2^ M.D. Ph.D., *Cheli Melzer-Cohen^3^ M.Sc., Aliza Rozenberg^1,2^ MA, Ilan Yanuv^1,2^ M.Sc., Gabriel Chodick^3,4^ Ph.D., Avraham Karasik ^3,5^ M.D., Mikhail Kosiborod^6,7^ MD, Ofri Mosenzon^1,2^ M.D. M.Sc.

* These authors have equally contributed to this work

^1^ Faculty of Medicine, Hebrew University of Jerusalem, Israel

^2^ Diabetes Unit, Department of Endocrinology and Metabolism, Hadassah Medical Center, Jerusalem, Israel

^3^ Maccabi Institute for Research and Innovation, Maccabi Healthcare Services, Tel-Aviv, Israel

^4^ School of Public Health Sackler Faculty of Medicine Tel Aviv University Tel Aviv Israel

^5^ Tel Aviv University, Tel Aviv, Israel

^6^ Saint Luke’s Mid America Heart Institute, University of Missouri-Kansas City, Kansas, MO, USA

^7^ The George Institute for Global Health and University of New South Wales, Sydney, New South Wales, Australia

**Counts**:

Abstract: 337 words

Manuscript: 4173 words (excl. Acknowledgements, References and legends)

54 References

1 Table, 3 Figures

Appendix: 3 Tables; 4 Figures

**Author for correspondence:**

Ofri Mosenzon M.D., M.Sc.

The Diabetes Unit, Department of Endocrinology and Metabolism

Hadassah Ein Kerem Medical Center

P.O.B 12000

Jerusalem 9112001

Israel

**Supplementary methods**

Full list of variables used for propensity score development and matching

Age, sex, socioeconomic status (low, low-medium, medium, high), diabetes duration (<2, 2-<5, 5-<10, >10 years), frailty, index year, laboratory measurements (eGFR, HbA1c (<7, 7-<8, 8-<9, 9+ %), UACR (Urinary albumin BDL, <30, 30-<300, 300+ mg/g), BMI (<25, 25-30, 30-35, >35 kg/m^2^)), co-morbidities (diabetic kidney disease, neuropathy, retinopathy, myocardial infraction or PCI or CABG, cerebrovascular disease, HF, unstable angina, atrial fibrillation, peripheral artery disease, in hypertension registry (1), after bariatric surgery), GLAs (metformin, sulfonylurea, DPP-4 inhibitors, GLP-1 RA, meglitinides, thiazolidinedione, acarbose, short- acting insulin, intermediate insulin, basal insulin, pre-mix insulin) and other concomitant medications (statins, anti-hypertensive medications, receptor P2Y12 antagonists, low dose acetylic salicylic acid, other antiplatelets, warfarin, loop diuretic, aldosterone antagonists, weight loss medications). Importantly, eGFR-slope at baseline was not used for propensity-score matching.

**Supplementary figure 1: Risk for cardiovascular and kidney outcome in SGLT2i initiators compared to oGLAs in the entire cohort, during the OT follow up definition.**

Event rates are presented as number of events per 100 person years of follow up. In black – the unadjusted model; and in grey – the model adjusted to baseline eGFR (as continuous variable) and UACR (as categorical variable).

SGLT2i = sodium/glucose cotransporter-2 inhibitors; oGLAs = other glucose lowering agents; OT = on treatment; hHF = hospitalization for heart failure; ACM =all-cause mortality; MI = myocardial infract; eGFR = estimated glomerular filtration rate; ESKD = end stage kidney disease; ER = event rate.

**Supplementary figure 2: Risk for cardiovascular and kidney outcomes in SGLT2i initiators compared to oGLAs in low kidney risk populations, during the OT follow up definition.**

A – Cardiovascular outcomes

B – Kidney outcomes

Event rates are presented as number of events per 100 person-years of follow up. Low KDIGO risk is defined as eGFR>60 ml/min/1.73m^2^ and UACR<30 mg/g. For the low KDIGO risk and eGFR>90 ml/min/1.73 m^2^, the model was adjusted to baseline eGFR (as continuous variable) and UACR (as categorical variable). Outcome analysis of the urine albumin BDL category was only adjusted to baseline eGFR as continuous variable.

* BDL= Below detectable levels

SGLT2i = sodium/glucose cotransporter-2 inhibitors; oGLAs = other glucose lowering agents; OT = on treatment; hHF = hospitalization for heart failure; ACM =all-cause mortality; MI = myocardial infract; eGFR = estimated glomerular filtration rate; UACR = urinary albumin to creatinine ratio; KDIGO = kidney disease: improving global outcomes; ER =event rate.

**Supplementary figure 3: Risk for cardiovascular and kidney outcome in SGLT2i initiators compared to oGLAs in the entire cohort, during the sOT follow up definition.**

Event rates are presented as number of events per 100 person years of follow up. In black – the unadjusted model; and in grey – the model adjusted to baseline eGFR (as continuous variable) and UACR (as categorical variable).

SGLT2i = sodium/glucose cotransporter-2 inhibitors; oGLAs = other glucose lowering agents; sOT = strict on treatment; hHF = hospitalization for heart failure; ACM =all-cause mortality; MI = myocardial infract; eGFR = estimated glomerular filtration rate; ESKD = end stage kidney disease; ER = event rate.

**Supplementary figure 4: Risk for cardiovascular and kidney outcomes in SGLT2i initiators compared to oGLAs in low kidney risk populations, during the sOT follow up definition.**

A – Cardiovascular outcomes

B – Kidney outcomes

Event rates are presented as number of events per 100 person-years of follow up. Low KDIGO risk is defined as eGFR>60 ml/min/1.73m^2^ and UACR<30 mg/g. For the low KDIGO risk and eGFR>90 ml/min/1.73 m^2^, the model was adjusted to baseline eGFR (as continuous variable) and UACR (as categorical variable). Outcome analysis of the urine albumin BDL category was only adjusted to baseline eGFR as continuous variable.

* BDL= Below detectable levels

SGLT2i = sodium/glucose cotransporter-2 inhibitors; oGLAs = other glucose lowering agents; sOT = strict on treatment; hHF = hospitalization for heart failure; ACM =all-cause mortality; MI = myocardial infract; eGFR = estimated glomerular filtration rate; UACR = urinary albumin to creatinine ratio; KDIGO = kidney disease: improving global outcomes; ER =event rate.

**Supplementary table S1**

| **Covariates** | **Definition** |
| --- | --- |
| Type 2 diabetes mellitus | Included in MHS registry any-time prior to index date (2) |
| Socioeconomic status | A 1-10 scale of socioeconomic status (1-low, 10-high), including data from Israel Central Bureau of Statistics as well as updates on new settlements. |
| Established CVD history | Included in MHS registry any time prior to index date. Includes the following: Ischemic heart disease, cerebrovascular disease or peripheral vascular disease. (3) |
| Myocardial infarction/ CABG/ PCI with stent | Included in MHS registry any time prior to index date. (3) |
| Unstable angina | ICD-9 codes: 411.x |
| Angina pectoris | ICD-9 codes: 413.x, 414.x |
| Heart failure | Included in MHS registry any time prior to index date. (3) |
| Atrial fibrillation | Included in MHS registry any time prior to index date. (3) |
| Stroke | ICD-9 codes: 430.x-434.x, 436.x |
| Hemorrhagic | ICD-9 codes: 430.x-432.x |
| Ischemic | ICD-9 codes: 433.x, 434.x-436.x |
| Transitory ischemic attack | Included in MHS registry any time prior to index date. (3) |
| Peripheral artery disease | ICD-9 codes: 440.x, 441.x, 444.x |
| Bariatric surgery | CPT codes: 43644, 43842, 43845, 43848 |
| Diabetic kidney disease | ICD-9 codes: 250.4, 583.81, or urinary albumin to creatinine ratio (UACR)>100mg/g or eGFR<60 mL/min/1.73 m² |
| Diabetic eye complications | ICD-9 codes: 362.0, 362.01, 362.02, 362.07, 365.44, 366.41, 250.5 |
| Diabetic mono-/polyneuropathy | ICD-9 codes: 357.2, 250.6, 354.x, 355.x |
| Diabetic foot/Peripheral angiopathy | ICD-9 codes: 713.5, 250.7, 250.8; CPT codes: 35363, 35355, 35521, 35546, 35556, 35558, 35565, 35141. |
| Hypertension | Included in MHS registry any time prior to index date. (1) |
| Frailty | Hospitalized for at least 3 days within 1 year prior to index date |
| Cancer | Included in national cancer registry or in MHS cancer registry. (4) |
| Metformin | ATC codes: A10BA, A10BD07, A10BD08, A10BD10, A10BD11, A10BD15, A10BD20 |
| SGLT2 inhibitors | ATC codes: A10BD16, A10BD20, A10BK01, A10BK03, A10BD15, A10BD16, A10BD20, A10BD15, A10BD19, A10BD21 |
| Sulfonylureas | ATC codes: A10BB |
| DPP4 inhibitors | ATC codes: A10BH , A10BD07, A10BD08, A10BD10, A10BD11, A10BD19, A10BD21 |
| GLP-1 RA | ATC codes: A10BJ02, A10BJ03, A10BJ05, A10AE54, A10AE56 |
| Meglitinides | ATC codes: A10BX02, A10BX03 |
| Thiazolidinediones | ATC codes: A10BG, A10BD03 , A10BD04 , A10BD05 , A10BD06 |
| Acarbose | ATC codes: A10BF |
| Insulin | ATC codes: A10A |
| Short-acting | ATC codes: A10AB |
| Intermediate-acting (isophane) | ATC codes: A10AC |
| Long-acting | ATC codes: A10AE |
| Premixed insulin | ATC codes: A10AD |
| Low dose acetylic salicylic acid | ATC codes: B01 (excluding B01AB01) |
| Statins | ATC codes: C10AA , C10BA , C10BX |
| Antihypertensives |  |
| ACE inhibitors | ATC codes: C09A , C09B |
| ARB | ATC codes: C09C C09D |
| Dihydropyridines (calcium channel blockers) | ATC codes: C08 , C07FB , C09BB, C09DB |
| Low ceiling diuretics (thiazides) | ATC codes: C03A |
| Beta blockers | ATC codes: C07 |
| High ceiling diuretics (loop-diuretics) | ATC codes: C03C |
| Aldosterone antagonists | ATC codes: C03DA |
| Warfarin | ATC codes: B01AA03 |
| Receptor P2Y12 antagonists | ATC codes: B01AC04, B01AC22, B01AC24, |
| Other antiplatelets | ATC codes: B01AC07, B01AC09, B01AC11, B01AC13, B01AC16, B01AC17, B01AC21 |
| Weight loss drugs | ATC codes: A08A |
|  | |
| **Outcomes** | **Definition** |
| Hospitalization for heart failure | Hospitalization with ICD-9 codes: 428.x |
| Myocardial infraction | Hospitalization with ICD-9 codes: 410.x |
| Stroke | Hospitalization with ICD-9 codes: 430.x-434.x, 436.x |
| End stage kidney disease | eGFR<15 mL/min/1.73 m² or defined as ESKD in CKD registry (5) (eGFR<15 mL/min/1.73 m²; ICD-9 codes: 39.95, 54.98, V56.0x, V56.8x, V45.1x; CPT codes: 90935, 90945, 48554) |

**Table S2: Additional patients’ baseline characteristics post propensity-matching**

|  | | **Study group** | | |
| --- | --- | --- | --- | --- |
| **Characteristic** | **Level** | **SGLT2i (N=9219)** | **oGLAs (N=9219)** | **STD** |
| **Demographic characteristics** |  |  |  |  |
| Years in diabetes registry, mean (SD) |  | 11.0 ( 5.3 ) | 10.8 ( 5.2 ) | 0.04 |
| Calendar year of Index date, n (%) | 2015 | 2103 (22.8%) | 2114 (22.9%) | 0.00 |
|  | 2016 | 3155 (34.2%) | 3162 (34.3%) |  |
|  | 2017 | 2990 (32.4%) | 2951 (32.0%) |  |
|  | 2018 | 971 (10.5%) | 992 (10.8%) |  |
| Socioeconomic status, mean (SD) |  | 6.0 ( 1.9 ) | 6.0 ( 1.8 ) | 0.00 |
| Current smokers, n (%) | No | 4115 (44.6%) | 3967 (43.0%) |  |
|  | Yes | 914 (9.9%) | 1058 (11.5%) | -0.05 |
|  | Missing | 4190 (45.4%) | 4194 (45.5%) |  |
| **Baseline measures** |  |  |  |  |
| BMI, n (%) | <25 kg/m² | 662 (7.2%) | 650 (7.1%) | 0.02 |
|  | 25-30 kg/m² | 2619 (28.4%) | 2619 (28.4%) |  |
|  | 30-35 kg/m² | 2766 (30.0%) | 2786 (30.2%) |  |
|  | 35+ kg/m² | 1845 (20.0%) | 1814 (19.7%) |  |
|  | Missing | 1327 (14.4%) | 1350 (14.6%) |  |
| Systolic blood pressure (mmHg) | Mean (SD) | 132.9 ( 15.3 ) | 133.0 ( 15.4 ) | -0.00 |
| Diastolic blood pressure (mmHg) | Mean (SD) | 76.8 ( 9.1 ) | 77.1 ( 9.3 ) | -0.04 |
| eGFR (mL/min/1.73 m²) | Mean (SD) | 88.2 ( 17.6 ) | 88.5 ( 19.3 ) | -0.02 |
| eGFR slope (mL/min/1.73 m² /year) | Mean (SD) | -1.1 ( 2.7 ) | -1.1 ( 3.0 ) | 0.00 |
| UACR ^$^ | Median (IQR) | 13 (0, 51) | 12 (0, 49) |  |
| HDL, mmol/L | Mean(SD) | 1.1 ( 0.3 ) | 1.1 ( 0.3 ) | -0.05 |
| LDL, mmol/L | Mean(SD) | 2.3 ( 0.9 ) | 2.4 ( 0.9 ) | -0.13 |
| **Baseline medications** |  |  |  |  |
| Low dose acetylic salicylic acid | n (%) | 4448 (48.2%) | 4504 (48.9%) | -0.01 |
| Statins | n (%) | 7417 (80.5%) | 7446 (80.8%) | -0.01 |
| Antihypertensives | n (%) | 7106 (77.1%) | 7106 (77.1%) | 0.00 |
| Dihydropyridines (calcium channel blockers) | n (%) | 1943 (21.1%) | 2010 (21.8%) | -0.02 |
| Low ceiling diuretics (thiazides) | n (%) | 539 (5.8%) | 539 (5.8%) | 0.00 |
| High ceiling diuretics (loop-diuretics) | n (%) | 572 (6.2%) | 541 (5.9%) | 0.01 |
| Warfarin | n (%) | 151 (1.6%) | 154 (1.7%) | -0.00 |
| Receptor P2Y12 antagonists | n (%) | 1087 (11.8%) | 1095 (11.9%) | -0.00 |
| Weight loss drugs | n (%) | 37 (0.4%) | 38 (0.4%) | -0.00 |
| **Medical history** |  |  |  |  |
| Unstable angina | n (%) | 1417 (15.4%) | 1437 (15.6%) | -0.01 |
| Angina pectoris | n (%) | 859 (9.3%) | 878 (9.5%) | -0.01 |
| Coronary heart failure | n (%) | 344 (3.7%) | 307 (3.3%) | 0.02 |
| Atrial fibrillation | n (%) | 439 (4.8%) | 437 (4.7%) | 0.00 |
| Bariatric surgery | n (%) | 261 (2.8%) | 264 (2.9%) | -0.00 |
| Diabetic kidney disease | n (%) | 2409 (26.1%) | 2215 (24.0%) | 0.05 |
| Diabetic eye complications | n (%) | 2104 (22.8%) | 2105 (22.8%) | -0.00 |
| Diabetic mono-/polyneuropathy | n (%) | 3533 (38.3%) | 3572 (38.7%) | -0.01 |
| Diabetic foot/Peripheral angiopathy | n (%) | 818 (8.9%) | 839 (9.1%) | -0.01 |
| Hypertension | n (%) | 6277 (68.1%) | 6288 (68.2%) | -0.00 |
| Frailty | n (%) | 866 (9.4%) | 834 (9.0%) | 0.01 |
| Cancer | n (%) | 1241 (13.5%) | 1288 (14.0%) | -0.01 |

Legends:

^$^ STD was not calculated for UACR as a continuous variable, because it does not follow normal distribution.

**Table S3: Distribution of index medications post-match and by follow up definitions**

|  | | **Exposure years (mean (SD), [median])** | | | **Pearson years** | | | |  |
| --- | --- | --- | --- | --- | --- | --- | --- | --- | --- |
|  | **n (%)** | **OT** | **ITT** | **sOT** | **OT** | **ITT** | **sOT** | |  |
| **SGLT2i** | **9219 (50.0%)** | **0.9 (0.8), [0.5]** | **1.7 (0.9), [1.7]** | **0.8 (0.8), [0.5]** | **8126.4 (100.0%)** | **15583.6 (100.0%)** | | **7303.7 (100.0%)** | |
| Dapagliflozin | 3137 (34.0%) | 1.1 (1.0), [0.7] | 2.3 (0.8), [2.6] | 1.0 (1.0), [0.6] | 3559.1 (43.8%) | 7233.1 (46.4%) | | 3141.1 (43.0%) | |
| Empagliflozin | 6082 (66.0%) | 0.8 (0.7), [0.5] | 1.4 (0.7), [1.4] | 0.7 (0.6), [0.4] | 4567.3 (56.2%) | 8350.4 (53.6%) | | 4162.6 (57.0%) | |
| **oGLAs** | **9219 (50.0%)** | **0.7 (0.7), [0.4]** | **1.7 (0.9), [1.7]** | **0.6 (0.7), [0.3]** | **6697.8 (100.0%)** | **15651.7 (100.0%)** | | **5672.8 (100.0%)** | |
| Acarbose | 86 (0.9%) | 0.6 (0.8), [0.2] | 2.1 (1.0), [2.4] | 0.6 (0.7), [0.2] | 53.7 (0.8%) | 182.4 (1.2%) | | 48.6 (0.9%) | |
| DPP4i | 2864 (31.1%) | 0.8 (0.8), [0.5] | 1.6 (0.9), [1.5] | 0.7 (0.7), [0.5] | 2392.2 (35.7%) | 4565.0 (29.2%) | | 2107.7 (37.2%) | |
| GLP1-RA | 1547 (16.8%) | 0.9 (0.8), [0.6] | 1.9 (0.9), [1.9] | 0.7 (0.7), [0.4] | 1398.8 (20.9%) | 2881.9 (18.4%) | | 1072.4 (18.9%) | |
| Insulin | 1023 (11.1%) | 0.4 (0.5), [0.2] | 1.7 (0.9), [1.8] | 0.3 (0.4), [0.2] | 404.0 (6.0%) | 1769.2 (11.3%) | | 306.7 (5.4%) | |
| Meglitinides | 610 (6.6%) | 0.6 (0.6), [0.4] | 1.7 (0.9), [1.8] | 0.5 (0.6), [0.3] | 364.7 (5.4%) | 1039.6 (6.6%) | | 320.1 (5.6%) | |
| Metformin | 1678 (18.2%) | 0.7 (0.7), [0.4] | 1.5 (0.9), [1.4] | 0.6 (0.6), [0.3] | 1110.0 (16.6%) | 2570.5 (16.4%) | | 983.9 (17.3%) | |
| Sulfonylureas | 890 (9.7%) | 0.7 (0.7), [0.4] | 1.8 (0.9), [1.9] | 0.6 (0.6), [0.3] | 594.5 (8.9%) | 1641.7 (10.5%) | | 519.9 (9.2%) | |
| TZDs | 521 (5.7%) | 0.7 (0.8), [0.4] | 1.9 (0.9), [2.1] | 0.6 (0.7), [0.3] | 379.9 (5.7%) | 1001.4 (6.4%) | | 313.5 (5.5%) | |

**Abbreviations**: PP4i – Dipeptidyl peptidase-4 inhibitor; GLP1-RA – Glucagon-like peptide-1 receptor agonists; ITT– intention to treat; oGLAs – other glucose lowering agents; OT – on treatment; SGLT2i – Sodium-glucose cotransporter 2 inhibitors; sOT – strict on treatment; TZDs – Thiazolidinediones

References

1. Weitzman D, Chodick G, Shalev V, Grossman C, Grossman E. Prevalence and factors associated with resistant hypertension in a large health maintenance organization in Israel. Hypertension. 2014 Sep;64(3):501–507.

2. Chodick G, Heymann AD, Shalev V, Kookia E. The epidemiology of diabetes in a large Israeli HMO. Eur J Epidemiol. 2003;18(12):1143–1146.

3. Shalev V, Chodick G, Goren I, Silber H, Kokia E, Heymann AD. The use of an automated patient registry to manage and monitor cardiovascular conditions and related outcomes in a large health organization. Int J Cardiol. 2011 Nov 3;152(3):345–349.

4. Israel National Cancer Registry, Israel Center for Disease Control, Ministry of Health [Internet]. 2018 [cited 2021 May 6]. Available from: https://www.health.gov.il/English/MinistryUnits/HealthDivision/Icdc/Icr/Pages/default.aspx

5. Coresh J, Turin TC, Matsushita K, Sang Y, Ballew SH, Appel LJ, et al. Decline in estimated glomerular filtration rate and subsequent risk of end-stage renal disease and mortality. JAMA. 2014 Jun 25;311(24):2518–2531.
