# Supplementary figures and images for: Cardiorenal outcomes with sodium/glucose cotransporter-2 inhibitors in patients with type 2 diabetes and low kidney risk: real world evidence
Source: Cardiovasc Diabetol. 2021 Aug 18;20:169. doi: 10.1186/s12933-021-01362-y (PMC8375057; doi:10.1186/s12933-021-01362-y)

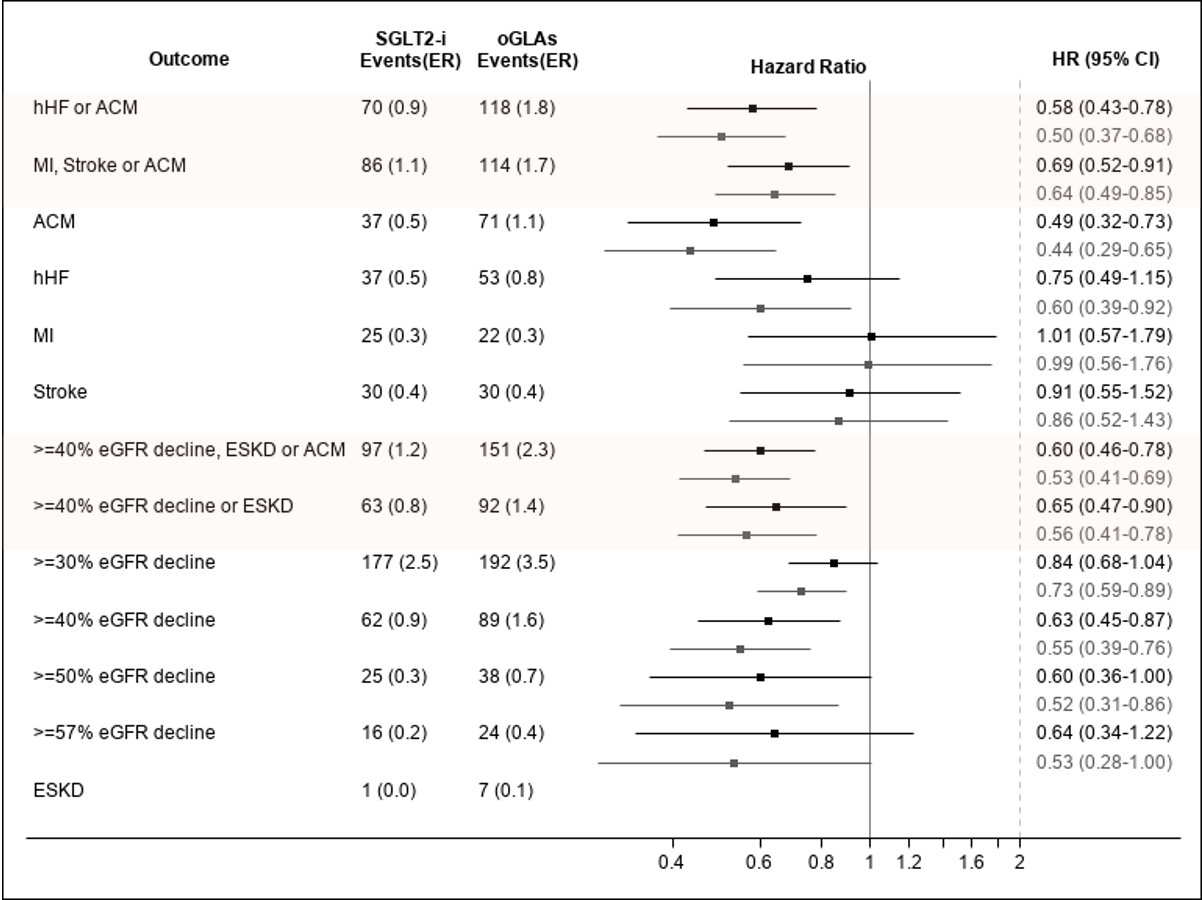

Supplement: Supplementary file 2 — Additional file 2:Figure S1. Risk for cardiovascular and kidney outcome in SGLT2i initiators compared to oGLAs in the entire cohort, during the OT follow up definition. Event rates are presented as number of events per 100 person years of follow up. In black—the unadjusted model; and in grey—the model adjusted to baseline eGFR (as continuous variable) and UACR (as categorical variable). SGLT2i = sodium/glucose cotransporter-2 inhibitors; oGLAs = other glucose lowering agents; OT = on treatment; hHF = hospitalization for heart failure; ACM =all-cause mortality; MI = myocardial infract; eGFR = estimated glomerular filtration rate; ESKD = end stage kidney disease; ER = event rate. [file 12933_2021_1362_MOESM2_ESM.tif]

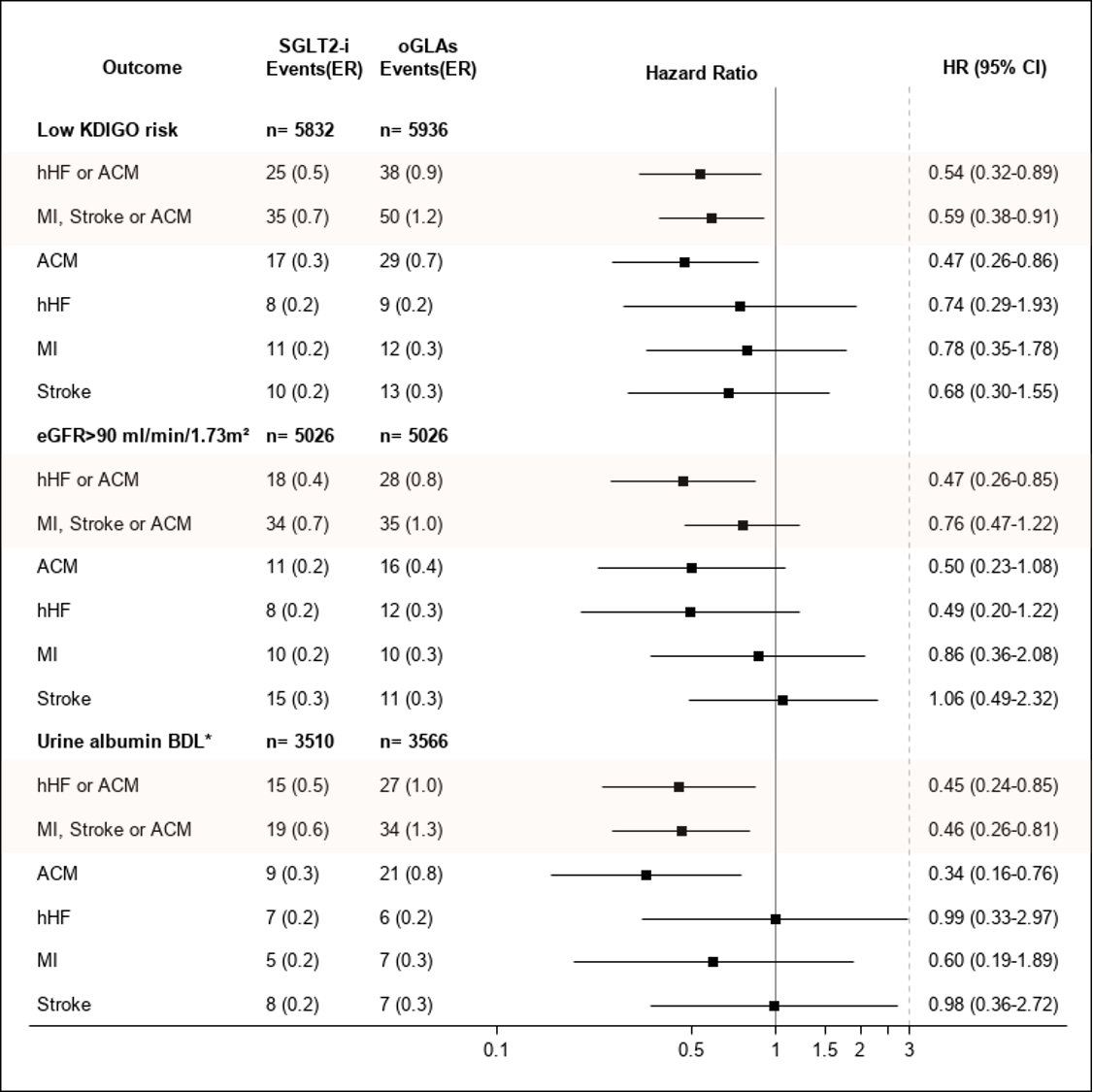

Supplement: Supplementary file 3 — Additional file 3: Figure S2. Risk for cardiovascular and kidney outcomes in SGLT2i initiators compared to oGLAs in low kidney risk populations, during the OT follow up definition. A Cardiovascular outcomes. B Kidney outcomes. Event rates are presented as number of events per 100 person-years of follow up. Low KDIGO risk is defined as eGFR>60 ml/min/1.73 m2 and UACR<30 mg/g. For the low KDIGO risk and eGFR>90 ml/min/1.73 m2, the model was adjusted to baseline eGFR (as continuous variable) and UACR (as categorical variable). Outcome analysis of the urine albumin BDL category was only adjusted to baseline eGFR as continuous variable. * BDL= Below detectable levels. SGLT2i = sodium/glucose cotransporter-2 inhibitors; oGLAs = other glucose lowering agents; OT = on treatment; hHF = hospitalization for heart failure; ACM =all-cause mortality; MI = myocardial infract; eGFR = estimated glomerular filtration rate; UACR = urinary albumin to creatinine ratio; KDIGO = kidney disease: improving global outcomes; ER =event rate. [file 12933_2021_1362_MOESM3_ESM.tif]

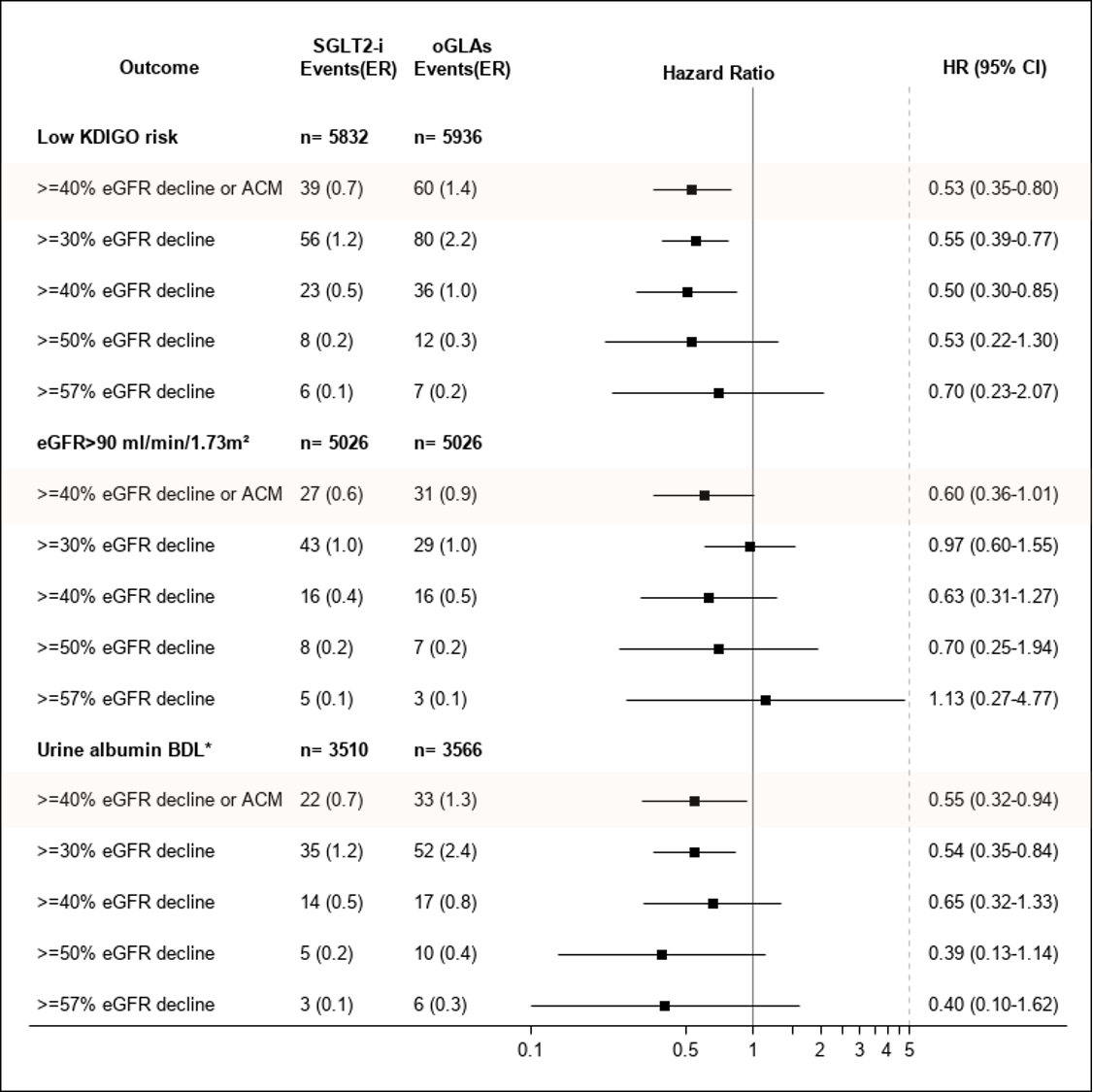

Supplement: Supplementary file 4 — Additional file 4: Figure S2. Risk for cardiovascular and kidney outcomes in SGLT2i initiators compared to oGLAs in low kidney risk populations, during the OT follow up definition. A Cardiovascular outcomes. B Kidney outcomes. Event rates are presented as number of events per 100 person-years of follow up. Low KDIGO risk is defined as eGFR>60 ml/min/1.73 m2 and UACR<30 mg/g. For the low KDIGO risk and eGFR>90 ml/min/1.73 m2, the model was adjusted to baseline eGFR (as continuous variable) and UACR (as categorical variable). Outcome analysis of the urine albumin BDL category was only adjusted to baseline eGFR as continuous variable. * BDL= Below detectable levels. SGLT2i = sodium/glucose cotransporter-2 inhibitors; oGLAs = other glucose lowering agents; OT = on treatment; hHF = hospitalization for heart failure; ACM =all-cause mortality; MI = myocardial infract; eGFR = estimated glomerular filtration rate; UACR = urinary albumin to creatinine ratio; KDIGO = kidney disease: improving global outcomes; ER =event rate. [file 12933_2021_1362_MOESM4_ESM.tif]

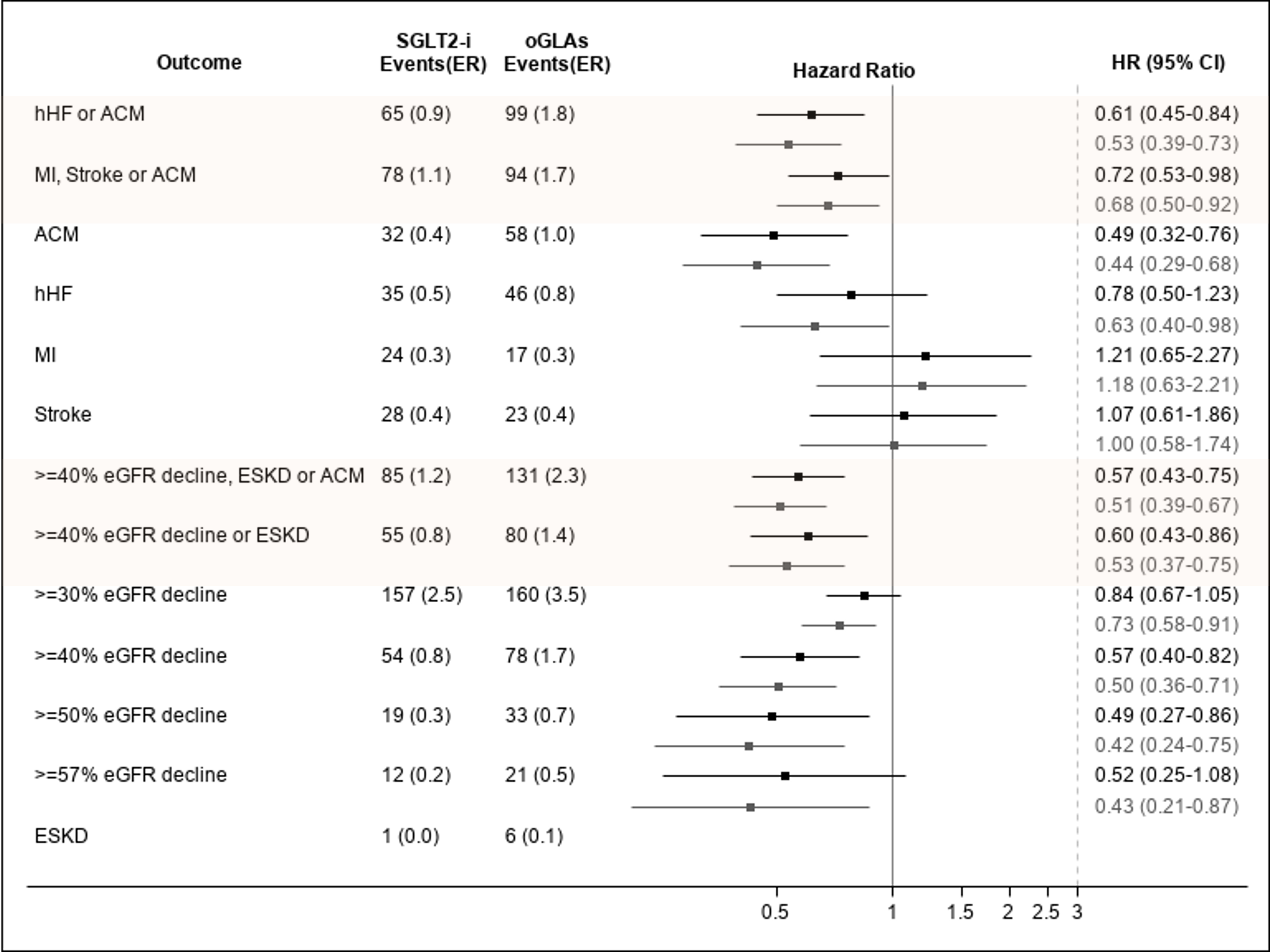

Supplement: Supplementary file 5 — Additional file 5: Figure S3. Risk for cardiovascular and kidney outcome in SGLT2i initiators compared to oGLAs in the entire cohort, during the sOT follow up definition. Event rates are presented as number of events per 100 person years of follow up. In black—the unadjusted model; and in grey—the model adjusted to baseline eGFR (as continuous variable) and UACR (as categorical variable). SGLT2i = sodium/glucose cotransporter-2 inhibitors; oGLAs = other glucose lowering agents; sOT = strict on treatment; hHF = hospitalization for heart failure; ACM =all-cause mortality; MI = myocardial infract; eGFR = estimated glomerular filtration rate; ESKD = end stage kidney disease; ER = event rate. [file 12933_2021_1362_MOESM5_ESM.tif]

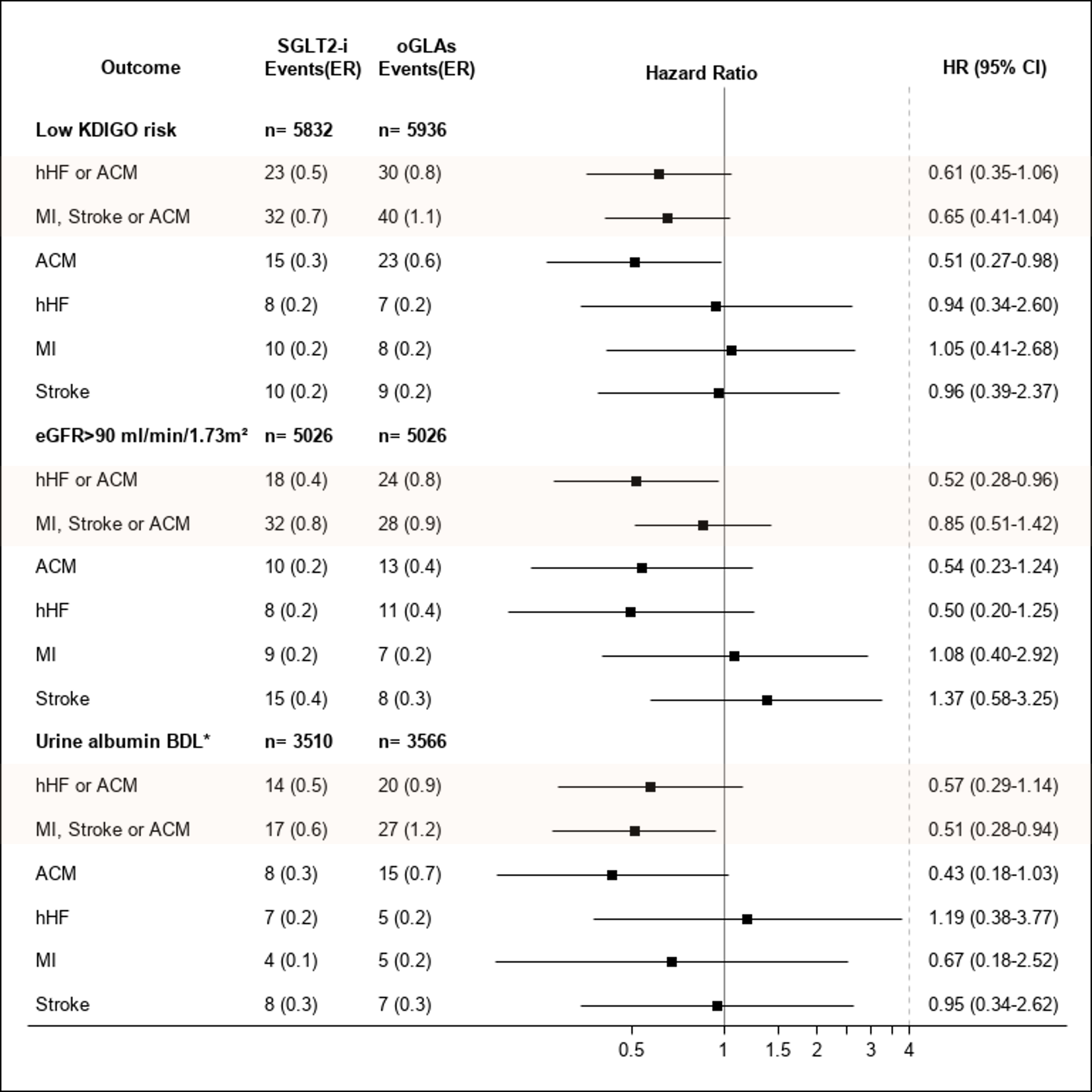

Supplement: Supplementary file 6 — Additional file 6: Figure S4. Risk for cardiovascular and kidney outcomes in SGLT2i initiators compared to oGLAs in low kidney risk populations, during the sOT follow up definition. A Cardiovascular outcomes. B Kidney outcomes. Event rates are presented as number of events per 100 person-years of follow up. Low KDIGO risk is defined as eGFR>60 ml/min/1.73 m2 and UACR<30 mg/g. For the low KDIGO risk and eGFR>90 ml/min/1.73 m2, the model was adjusted to baseline eGFR (as continuous variable) and UACR (as categorical variable). Outcome analysis of the urine albumin BDL category was only adjusted to baseline eGFR as continuous variable. *BDL= Below detectable levels. SGLT2i = sodium/glucose cotransporter-2 inhibitors; oGLAs = other glucose lowering agents; sOT = strict on treatment; hHF = hospitalization for heart failure; ACM =all-cause mortality; MI = myocardial infract; eGFR = estimated glomerular filtration rate; UACR = urinary albumin to creatinine ratio; KDIGO = kidney disease: improving global outcomes; ER =event rate. [file 12933_2021_1362_MOESM6_ESM.tif]

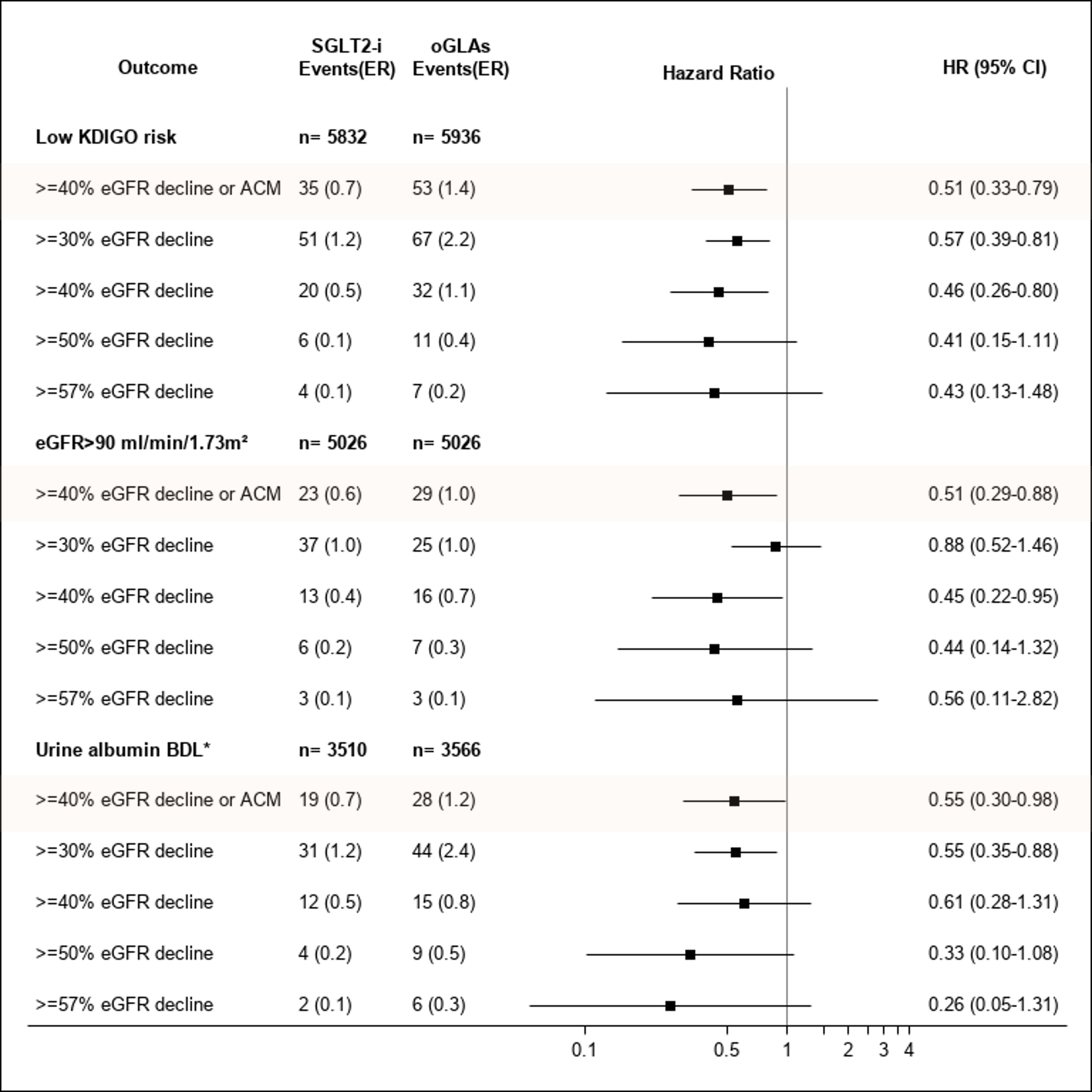

Supplement: Supplementary file 7 — Additional file 7: Figure S4. Risk for cardiovascular and kidney outcomes in SGLT2i initiators compared to oGLAs in low kidney risk populations, during the sOT follow up definition. A Cardiovascular outcomes. B Kidney outcomes. Event rates are presented as number of events per 100 person-years of follow up. Low KDIGO risk is defined as eGFR>60 ml/min/1.73 m2 and UACR<30 mg/g. For the low KDIGO risk and eGFR>90 ml/min/1.73 m2, the model was adjusted to baseline eGFR (as continuous variable) and UACR (as categorical variable). Outcome analysis of the urine albumin BDL category was only adjusted to baseline eGFR as continuous variable. *BDL= Below detectable levels. SGLT2i = sodium/glucose cotransporter-2 inhibitors; oGLAs = other glucose lowering agents; sOT = strict on treatment; hHF = hospitalization for heart failure; ACM =all-cause mortality; MI = myocardial infract; eGFR = estimated glomerular filtration rate; UACR = urinary albumin to creatinine ratio; KDIGO = kidney disease: improving global outcomes; ER =event rate. [file 12933_2021_1362_MOESM7_ESM.tif]
